# The MarR family regulator OsbR controls oxidative stress response, anaerobic nitrate respiration, and biofilm formation in *Chromobacterium violaceum*

**DOI:** 10.1186/s12866-021-02369-x

**Published:** 2021-11-04

**Authors:** Júlia A. Alves, Maristela Previato-Mello, Kelly C. M. Barroso, Tie Koide, José F. da Silva Neto

**Affiliations:** 1 Departamento de Biologia Celular e Molecular e Bioagentes Patogênicos, Faculdade de Medicina de Ribeirão Preto, Universidade de São Paulo, Ribeirão Preto, SP Brazil; 2 Departamento de Bioquímica e Imunologia, Faculdade de Medicina de Ribeirão Preto, Universidade de São Paulo, Ribeirão Preto, SP Brazil

**Keywords:** *Chromobacterium violaceum*, MarR family regulator, OsbR regulon, Transcriptome analysis, Oxidative stress, Biofilm formation, Nitrate respiration

## Abstract

**Background:**

*Chromobacterium violaceum* is an environmental opportunistic pathogen that causes rare but deadly infections in humans. The transcriptional regulators that *C. violaceum* uses to sense and respond to environmental cues remain largely unknown.

**Results:**

Here, we described a novel transcriptional regulator in *C. violaceum* belonging to the MarR family that we named OsbR (oxidative stress response and biofilm formation regulator). Transcriptome profiling by DNA microarray using strains with deletion or overexpression of *osbR* showed that OsbR exerts a global regulatory role in *C. violaceum*, regulating genes involved in oxidative stress response, nitrate reduction, biofilm formation, and several metabolic pathways. EMSA assays showed that OsbR binds to the promoter regions of several OsbR-regulated genes, and the in vitro DNA binding activity was inhibited by oxidants. We demonstrated that the overexpression of *osbR* caused activation of *ohrA* even in the presence of the repressor OhrR, which resulted in improved growth under organic hydroperoxide treatment, as seem by growth curve assays. We showed that the proper regulation of the *nar* genes by OsbR ensures optimal growth of *C. violaceum* under anaerobic conditions by tuning the reduction of nitrate to nitrite. Finally, the *osbR* overexpressing strain showed a reduction in biofilm formation, and this phenotype correlated with the OsbR-mediated repression of two gene clusters encoding putative adhesins.

**Conclusions:**

Together, our data indicated that OsbR is a MarR-type regulator that controls the expression of a large number of genes in *C. violaceum*, thereby contributing to oxidative stress defense (*ohrA*/*ohrR*), anaerobic respiration (*narK1K2* and *narGHJI*), and biofilm formation (putative RTX adhesins).

**Supplementary Information:**

The online version contains supplementary material available at 10.1186/s12866-021-02369-x.

## Background

*Chromobacterium violaceum* is a Gram-negative betaproteobacterium found in tropical and subtropical ecosystems around the world, primarily in water and soil samples [1, 2]. *C. violaceum* strains produce several enzymes and secondary metabolites with biotechnological interest, including quitinases, hydrogen cyanide, siderophores, the antitumoral depsipeptide FR901228, and antibiotics, such as violacein. The purple pigment violacein has in vitro activity against eukaryotic cells and Gram-positive bacteria [1, 3]. From the environmental reservoir, *C. violaceum* can cause rare but deadly opportunistic infections in humans and other animals, entering the hosts mainly through skin lesions. The resulting disease manifests as fatal septicemia and abscesses in the lung and liver [2, 4]. Similar to other environmental pathogens, *C. violaceum* has complex regulatory systems and versatile metabolic capacities to survive under several stress conditions [5–7]. For example, genome sequence analyses indicate that *C. violaceum* possesses a nitrate reductase (Nar) to reduce nitrate to nitrite to obtain energy under anaerobic conditions [5, 6]. However, the transcriptional regulators involved in *C. violaceum* adaptation to diverse environments remain poorly investigated.

The MarR (multiple antibiotic resistance regulator) family of transcriptional regulators, widespread in bacteria and archaea, includes proteins involved in several bacterial processes, such as antibiotic resistance, virulence control, oxidative stress response, and catabolism of aromatic compounds [8–13]. Many MarR-type regulators act as transcriptional repressors that dissociate from DNA upon binding of small ligands or oxidation of conserved Cys residues [10–12]. One prototypical redox-sensing MarR regulator is OhrR, a Cys-based redox sensor that, upon oxidation by organic hydroperoxides, releases the transcription of *ohrA*, encoding a Cys-based peroxidase [14–17]. Other redox-sensing MarR regulators control large regulons and multiple bacterial responses in addition to antioxidant defense. For instance, OspR of *Pseudomonas aeruginosa*, an OhrR homolog, regulates pigment production, antibiotic resistance, virulence, and antioxidant enzymes [18, 19]; MgrA of *Staphylococcus aureus* is a global regulator of virulence, antibiotic resistance, biofilm, and clumping [20, 21]. Several homologs of OspR and MgrA have been characterized, including SarZ of *S. aureus*, MosR of *Mycobacterium tuberculosis,* AbfR of *Staphylococcus epidermidis*, BmoR of *Bacteroides fragilis*, and AsrR of *Enterococcus faecium* [22–26]. The homolog pairs OhrR/OspR in *P. aeruginosa* and MgrA/SarZ in *S. aureus* control distinct and overlapping functions [18, 22].

Based on the genome sequence, *C. violaceum* has more than two hundred predicted transcriptional regulators grouped in several families, including the MarR family with at least fifteen members [6]. However, only a few transcriptional regulators have been characterized in *C. violaceum*. The master regulator CilA activates most genes from the *Chromobacterium* pathogenicity islands 1 and 1a (Cpi-1/−1a) that encode a type III secretion system required for virulence [27, 28]. The iron-sensing regulator Fur controls several traits, including antioxidant defense, siderophore production, and virulence [29]. Recently, we described the regulons of the MarR-type regulators OhrR and EmrR in *C. violaceum* [30, 31]. We have shown that OhrR affects *C. violaceum* virulence and is involved in resistance to organic hydroperoxides by regulating *ohrA* [30, 32], while EmrR confers antibiotic resistance by regulating the MFS-type efflux pump EmrCAB [31]. In this work, we described a novel MarR family transcriptional regulator in *C. violaceum*, CV_3905, which we named OsbR (oxidative stress response and biofilm regulator). Transcriptome and phenotype analyses revealed that OsbR exerts a global regulatory role in *C. violaceum*, contributing to oxidative stress defense, anaerobic respiration, and biofilm formation. Our results also indicate a double regulation of OhrR and OsbR on the *ohrR/ohrA* system for fine-tuning the organic hydroperoxide response.

## Results and discussion

### Identification of *C. violaceum* OsbR (CV_3905) as an OspR/MgrA homolog

**Fig. 1:**
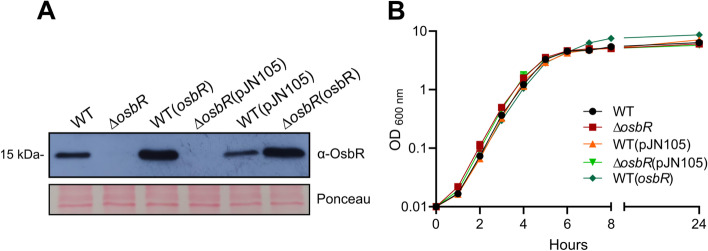
Construction and analyses of *C. violaceum* strains with deletion (∆*osbR*) or overexpression (WT(*osbR*)) of *osbR*. **a** The levels of OsbR in the indicated strains were analyzed by western blot using a polyclonal anti-OsbR antiserum. Equal protein loading was confirmed by Ponceau staining. Full-length blot and Ponceau-stained membrane are presented in Additional file 3, Fig. S3. **b** Growth curves of the indicated strains in LB medium. All strains showed a similar growth profile

The MarR-type regulators OspR of *P. aeruginosa*, MgrA of *S. aureus*, and their homologs in other bacteria, such as AbfR of *S. epidermidis* and MosR of *M. tuberculosis*, control multiple and complex bacterial phenotypes [19, 21, 23, 25]. To identify the OspR/MgrA homolog in *C. violaceum*, we performed BLASTP analyses with OspR or MgrA against the proteome derived from the genome sequence of *C. violaceum* ATCC 12472, using default parameters. The search yielded a single hit in *C. violaceum*, OhrR, which showed 34 and 47% identity with MgrA and OspR, respectively. Considering that we have previously characterized OhrR in *C. violaceum* [30, 32], we performed a subsequent search using the Position-Specific Iteractive BLAST (PSI-BLAST), which uses a position-specific score matrix (PSSM) to found distantly related proteins. In addition to OhrR, this search found as a second hit CV_3905, which shared 30% identity with MgrA (E value 6e-08 and cover 58%) and 35% identity with OspR (E value 2e-07 and cover 52%). Based on transcriptome and phenotype characterization presented in this work, we named CV_3905 as OsbR. The OsbR protein harbors two cysteine residues located at positions 55 and 133. Multiple sequence alignment revealed that the Cys55 residue of OsbR is conserved among closely related unstudied proteins. Still, it does not align with the reactive conserved Cys residues of well-characterized redox-sensing OspR/MgrA homologs (Additional file 1, Fig. S1). To characterize OsbR in *C. violaceum*, we constructed a null mutant strain ∆*osbR* and an overexpressing strain WT(*osbR*). The absence of OsbR in ∆*osbR* and the high levels of OsbR in WT(*osbR*) were confirmed by western blot (Fig. 1a). All strains presented the same growth pattern as the WT strain in the LB medium (Fig. 1b).

### Transcriptome analyses reveal a large and diverse OsbR regulon

**Fig. 2:**
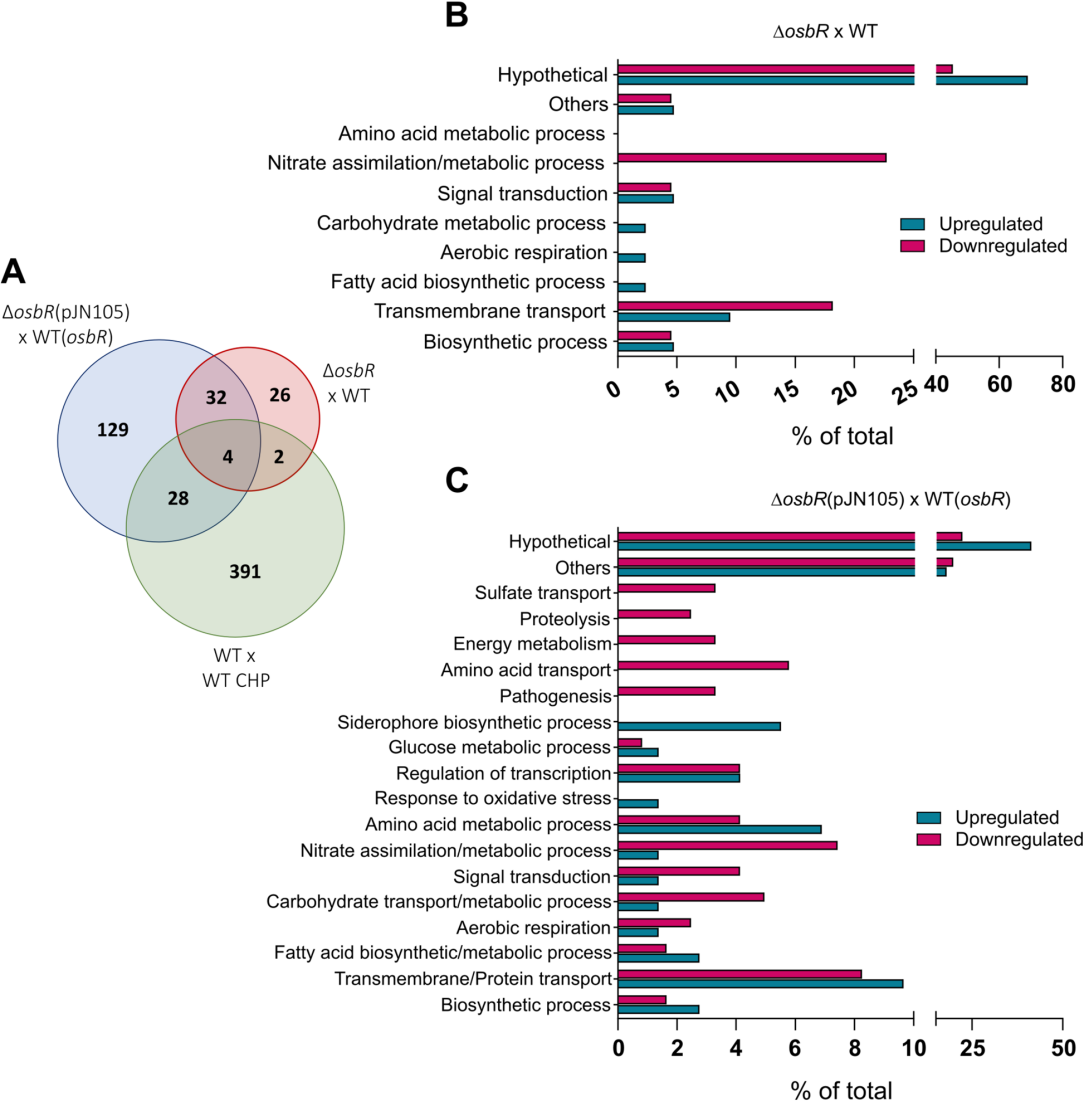
Microarray analyses revealed that ObsR regulates many genes involved in several biological processes. **a** Venn diagrams comparing the differentially expressed genes from the microarray analyses of this work (*osbR* strains) with published data (WT treated with the oxidant CHP). **b, c** Functional categorization by Gene Ontology (GO) of the differentially expressed genes from the comparison wild-type versus ∆*osbR* (**b**) and WT(*osbR*) versus ∆*osbR*(pJN105) (**c**)

To identify the OsbR regulon in *C. violaceum*, we performed two sets of DNA microarray analyses using, in both cases, three biological replicates of mid-log phase bacteria. Comparison of the transcriptome profiles of WT with ∆*osbR* revealed 65 differentially expressed genes (43 downregulated and 21 upregulated genes in ∆*osbR*) (Additional file 2, Table S1). In comparison, the transcriptome profiles of WT(*osbR*) versus ∆*osbR*(pJN105) showed a higher number of differentially expressed genes (72 downregulated and 121 upregulated genes in ∆*osbR*) (Additional file 2, Table S2) and more robust differences in the fold-change, including in the genes common to the two datasets (Fig. 2a, Additional file 2, Tables S1 and S2). Interesting, a subset of these genes, including the *ohrA*/*ohrR* system (organic hydroperoxide detoxification)*,* the *narGHJI* operon (nitrate reduction), and the *cbaABECF* operon (siderophore production) (Fig. 2a, Additional file 2, Table S3), were previously identified as members of the cumene hydroperoxide stimulon in *C. violaceum* [30], indicating a link between OsbR and oxidative stress (see below).

**Fig. 3:**
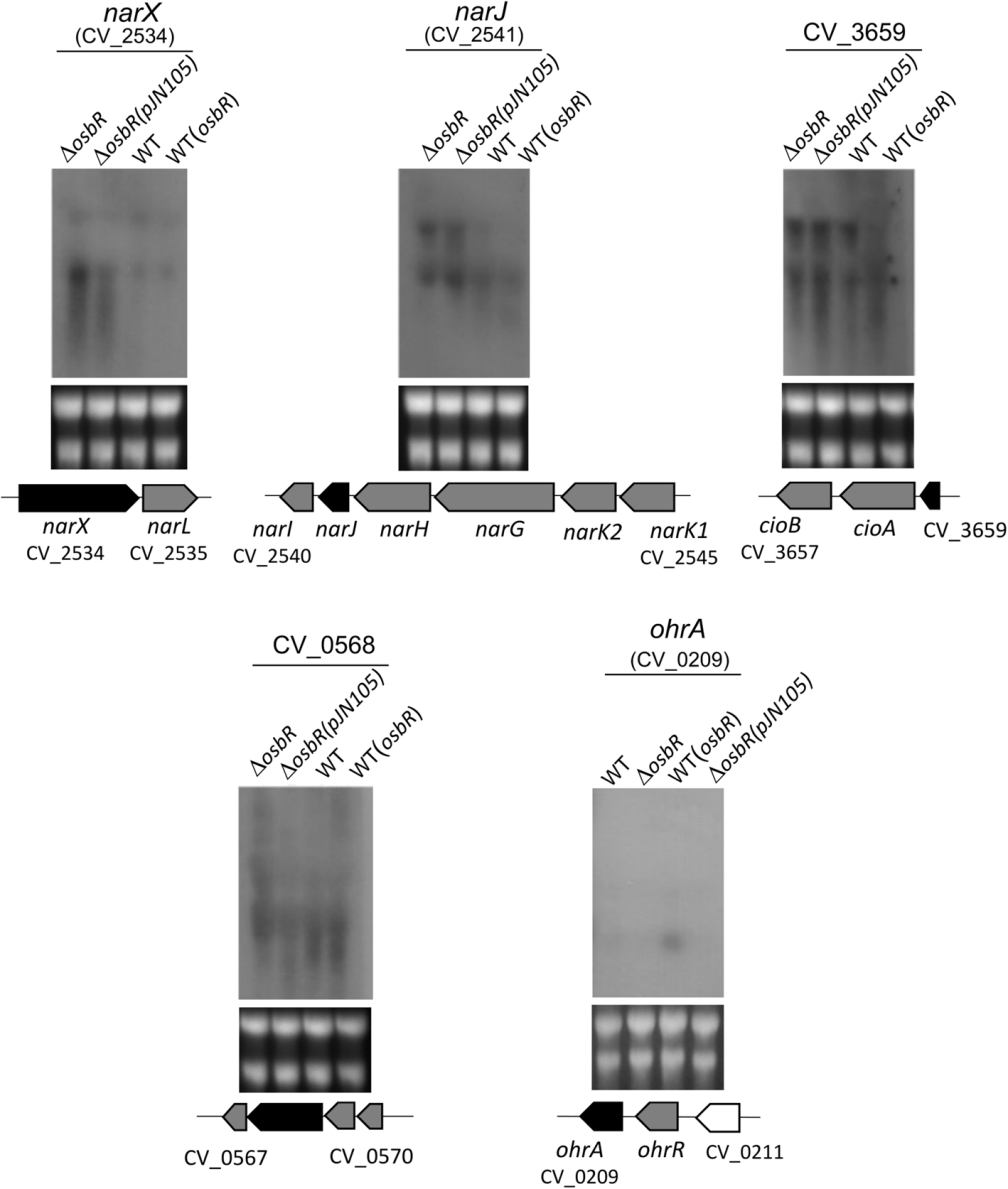
Validation by Northern blot of selected genes regulated by OsbR. RNA samples from the indicated *C. violaceum* strains (the same strains used in the microarray analyses) were transferred to a membrane and hybridized with specific radiolabeled probes for the selected genes (indicated by black arrows in the gene maps). The selected genes were repressed (*narX*, *narJ*, and CV_3659) or activated (CV_0568 and *ohrA*) by OsbR. Grey arrows indicate neighbor genes also differentially expressed according to microarray analyses. The rRNA were used as a loading control (Bottom gels). Full-length blot and RNA loading gel are presented in Additional file 3, Fig. S4

Functional categorization of the up- and downregulated genes identified in both microarray analyses reinforced that OsbR exerts a global transcriptional effect on the *C. violaceum* transcriptome with an increase in the number of genes and functional processes from the comparison WT versus ∆*osbR* (Fig. 2b) to the comparison WT(*osbR*) versus ∆*osbR*(pJN105) (Fig. 2c). Examples of biological processes affected by OsbR include the categories nitrogen metabolism, membrane transport, iron/siderophore, sulfur and amino acid metabolism, metabolic pathways, regulatory and signaling pathways, among others (Fig. 2b and c). To validate the microarray data, we performed a Northern blot assay probing the genes *narX* (*narXL* operon), *narJ* (*narK1K2GHJI* operon), CV_3659 (a MarR family regulator in an operon with *cioAB*), *ohrA* (*ohrR*/*ohrA*), and CV_0568 (anthranilate synthase in CV_0567-68-69-70 operon) (Fig. 3). The results from both microarrays and the Northern blot assay matched for all genes tested (*narX*, *narJ*, and CV_3659 were repressed by OsbR while CV_0558 and *ohrA* were activated by OsbR). The exception was CV_0568 in the comparison WT with ∆*osbR* since it was downregulated in ∆*osbR* in the microarray but not in the Northern blot assay.

### OsbR acts directly repressing and activating genes of its regulon

**Fig. 4:**
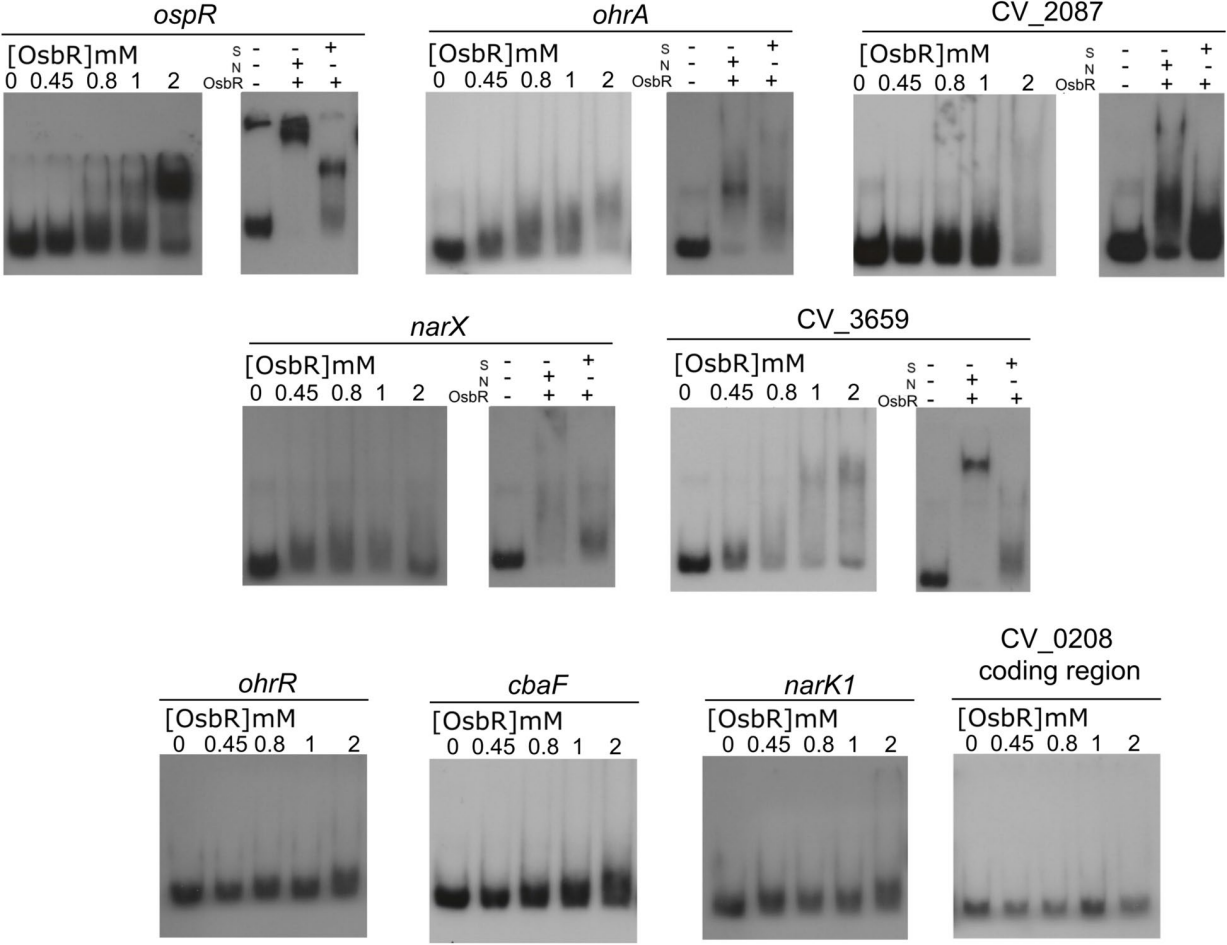
OsbR directly activates and represses genes from its regulon. EMSA assay was performed with radiolabeled probes corresponding to the promoter regions of the indicated genes incubated with increasing concentrations of OsbR. The coding region of CV_0208 was used as a negative control. Competition assays were performed with an excess of specific (S) or unspecified (N) unlabeled fragments

Several MarR family regulators act as repressors by directly binding in the promoter regions of their target genes [10, 11]. To verify if OsbR interacts in vitro with its own promoter and the promoter region of OsbR-regulated genes, we purified the His-OsbR protein (Additional file 1, Fig. S2). In the EMSA assays, an increasing amount of His-OsbR was incubated with labeled DNA fragments of the promoter regions (Fig. 4). The DNA binding assay showed that OsbR binds in different concentrations to the promoter regions of *osbR, ohrA*, CV_2087, *narX*, and CV_3659. The specificity of these interactions was confirmed by competition assays, adding in the reactions an unspecific (N) or a specific (S) cold probe (Fig. 4, right panels). Interestingly, OsbR binds to the promoter of both repressed (*narX* and CV_3659) and activated (*ohrA,* CV_2087) genes, suggesting a dual role of this regulator as repressor and activator. No band-shift was observed to the promoters of *narK1, ohrR, cbaF* and to the negative control (the coding region of CV_0208) (Fig. 4), indicating that these genes are indirectly regulated by OsbR. Considering that many transcriptional regulators are members of the OsbR regulon (Fig. 2c, Additional file 2, Table S2), it is tempting to propose that OsbR has its global regulatory role amplified by regulating other transcriptional regulators.

### OsbR responds to oxidation and has an antioxidant role

**Fig. 5:**
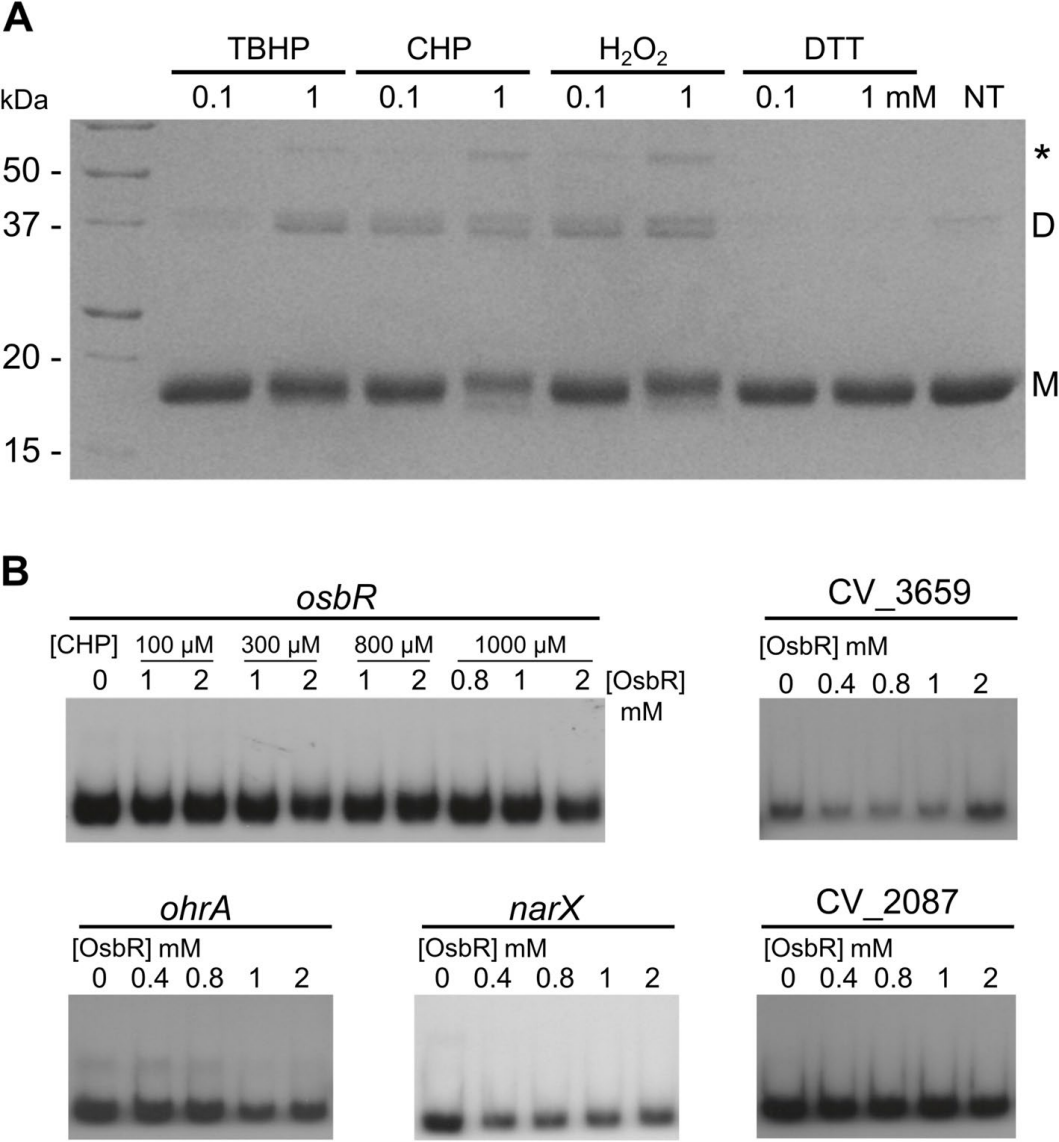
OsbR oxidation causes dimerization and inhibition of its DNA binding activity. **a** Formation of covalent OsbR dimers under oxidative stress in vitro. After 2 hours of exposure to oxidants or DTT, the His-OsbR protein was analyzed by non-reducing SDS-PAGE. NT refers to nontreated protein. Monomeric and dimeric forms are indicated by M and D, respectively. Asterisk indicates a third unknown multimeric form. **b** EMSA assay with oxidized OsbR. Prior to incubation with the DNA probes, His-OsbR was oxidized with 1 mM CHP for 1 hour (for the *osbR* probe, OsbR was oxidized with the indicated CHP concentrations)

Considering that OsbR has two cysteine residues (Additional file 1, Fig. S1) and when overexpressed activates the genes *ohrA*/*ohrR* (Fig. 3, Fig. 4, Additional file 2, Table S2), which encode an organic hydroperoxide antioxidant system in *C. violaceum* [30, 32], we investigated whether OsbR dimerizes upon oxidation by intermolecular disulfide bond formation, a feature of many Cys-based redox sensing regulators [16, 17, 19, 23, 32]. Indeed, the dimerization of monomeric purified His-OsbR was verified in non-reducing SDS-PAGE gel when this protein was incubated with the oxidants *tert*-butyl hydroperoxide (TBHP), cumene hydroperoxide (CHP), and hydrogen peroxide (H_2_O_2_) at 0.1 and 1 mM, but not with the reductant agent dithiothreitol (DTT) (Fig. 5a). Accordingly, when pretreated with CHP, the oxidized OsbR did not bind to the promoter regions of *ohrA,* CV_2087*,* CV_3659*, narX,* and *osbR* (Fig. 5b). Although further investigation is required to define the role of the OsbR Cys residues on oxidation sensing, our data indicated that the DNA binding activity of OsbR is inhibited by its oxidation.

**Fig. 6:**
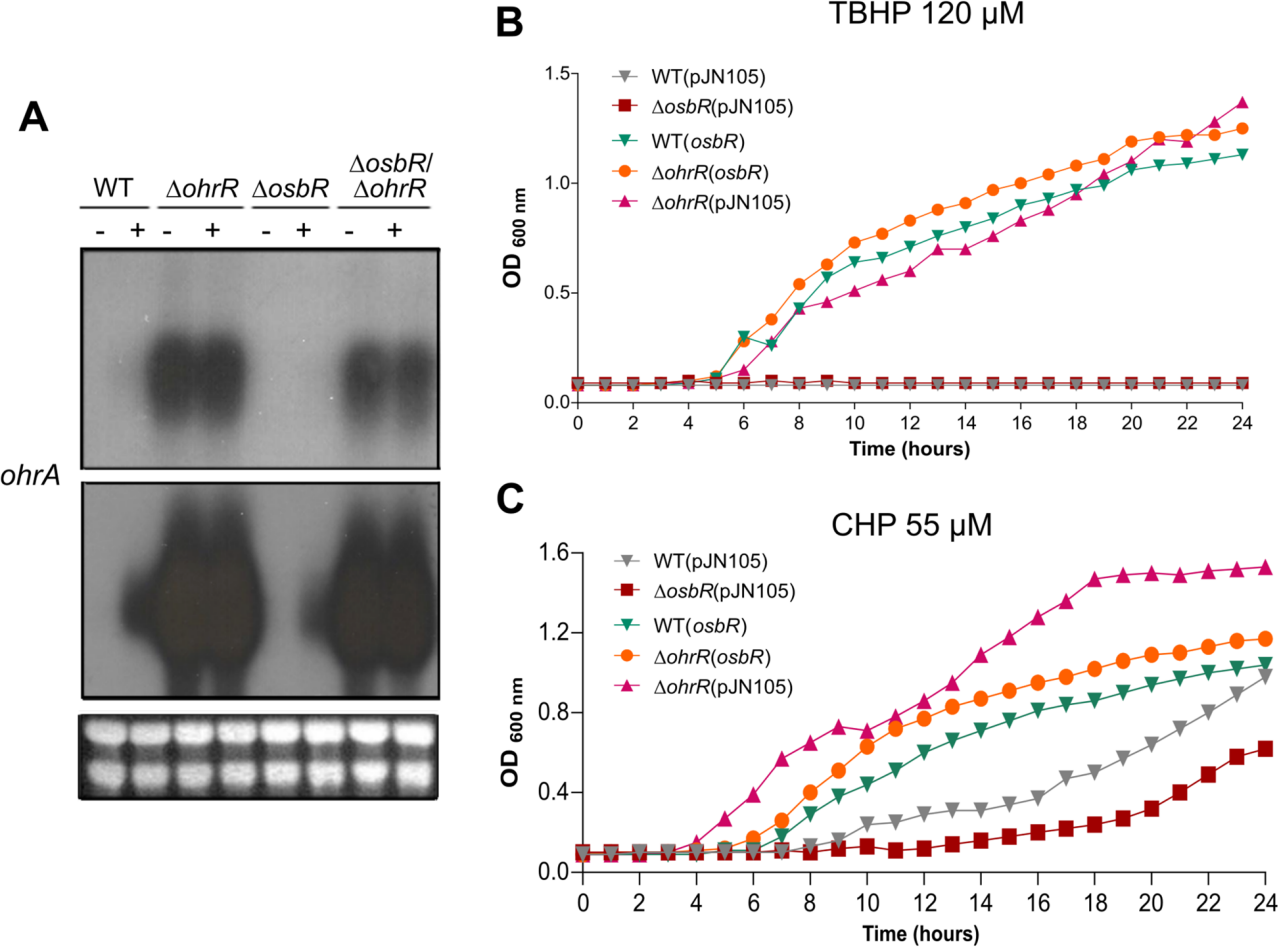
OsbR is required for fully *ohrA* expression and protection against oxidative stress. **a** Northern blot of *ohrA* using the indicated strains untreated (−) or treated (+) with 100 μM CHP for 10 min. The same film was exposed for 24 h (top) or 4 days (bottom). Ribosomal RNA loading is shown below. Full-length blot and RNA loading gel are presented in Additional file 3, Fig. S5. **b, c** Growth curves of different strains under oxidative stress. The indicated *C. violaceum* strains were grown in LB treated with either 120 μM TBHP **(b)** or 55 μM CHP **(c)**

Next, we investigated the relationship between OsbR and the OhrA/OhrR system on the response to organic hydroperoxides by Northern blot and growth curve assays (Fig. 6). The *ohrA* expression was increased in the wild-type strain under CHP treatment and reached the maximum levels even without treatment in the ∆*ohrR* mutant (Fig. 6a), confirming our previous data that OhrR is a repressor of *ohrA* [30, 32]. Importantly, deletion of *osbR* in both the wild-type and ∆*ohrR* backgrounds decreased the *ohrA* expression under CHP treatment (Fig. 6a), suggesting that the maximum *ohrA* expression requires both OhrR derepression and OsbR activation. Indeed, overexpression of OsbR caused activation of *ohrA* even in the absence of oxidative stress (Fig. 3). Consistently with this regulatory model, the growth curves under treatment with the oxidants TBHP (Fig. 6b) and CHP (Fig. 6c) indicated that the strains with high *ohrA* levels (WT(*osbR*), ∆*ohrR*(*osbR*), and ∆*ohrR*) showed better growth than the wild-type and ∆*ospR* strains. Our results showing an interconnection of OhrR and OsbR on the regulation of *ohrA* in *C. violaceum* resemble the cross-regulation of OhrR and OspR on the *ohr* and *gpx* genes described in *P. aeruginosa* [18]. The OspR of *P. aeruginosa* is a homolog of OhrR that binds and represses *ohr* and can even functionally complement an *ohrR* mutant strain [18]. Despite OspR being a repressor of *ohr* and OsbR a putative activator of *ohrA*, both transcriptional regulators are responsive to organic hydroperoxides.

Among the genes upregulated by CHP oxidative stress and activated by OsbR overexpression (Fig. 2a, Additional file 2, Table S3), we highlight the *cbaABECF* operon in addition to the *ohrA* and *ohrR* genes. Recently, we determined that this operon encodes enzymes required for the production of a catecholate-type siderophore in *C. violaceum* [33]. The *E. coli* catecholate-type siderophore enterobactin has recently been shown to be involved in protection against oxidative stress via an iron-independent radical scavenging activity [34]. The fact that the *cbaABECF* operon is activated by OsbR like *ohrA* in *C. violaceum* could suggest a dual OsbR-mediated mechanism for protection against oxidative stress.

### OsbR regulates genes involved in anaerobic nitrate respiration in *C. violaceum*

Among the differentially expressed genes found in both microarray analyses and showing the highest upregulation in the ∆*osbR* mutant were those involved in nitrate respiration (Fig. 2, Additional file 2, Tables S1 and S2). These genes include the *narXL* operon encoding for a two-component system (NarXL) and the *narK1K2GHJI* operon encoding for nitrate/nitrite transporters (NarK1 and NarK2) and a respiratory nitrate reductase (NarGHJI). The OsbR-mediated repression of both operons was validated by Northern blot (Fig. 3). As the OsbR binding occurred in the promoter of the *narXL* operon but not in the promoter of *narK1K2GHJI* (Fig. 4), we propose that OsbR acts via NarXL to regulate the *narK1K2GHJI* operon in *C. violaceum*. Indeed, the NarXL is a well-characterized two-component system that senses the nitrate/nitrite levels to activate *narGHJI* and *narK* expression in *Escherichia coli* and *Pseudomonas stutzeri* [35, 36]. Consistently with our data, it has been demonstrated that other MgrA/OspR homologs, such as MosR in *M. tuberculosis* [25, 37] and RosR in *Corynebacterium glutamicum* [38] regulate *nar* genes associated with the nitrate reduction for growth under hypoxic and anaerobic conditions [39].

**Fig. 7:**
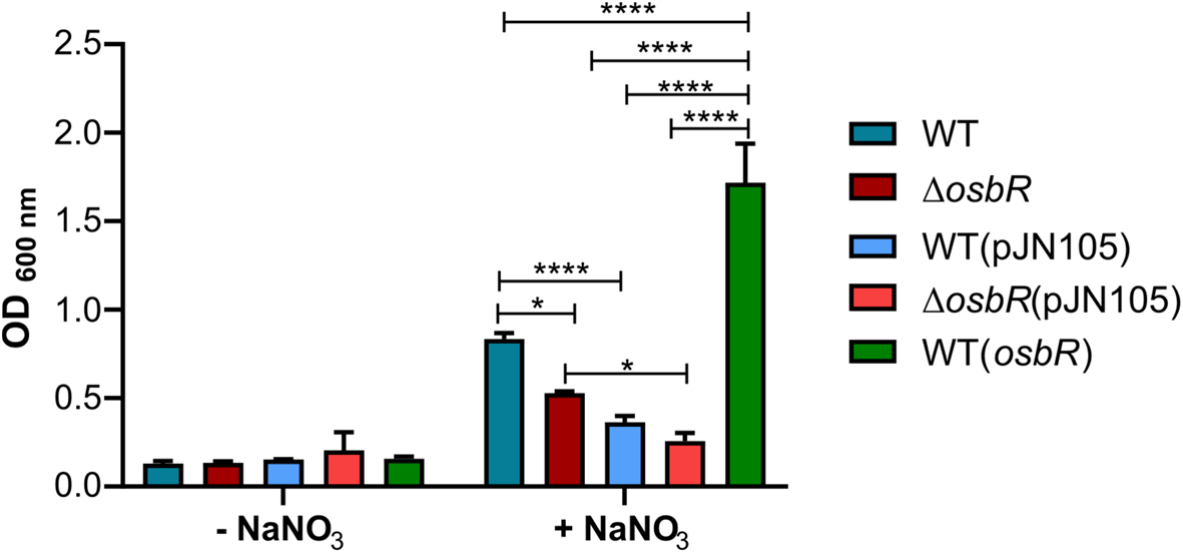
OsbR affects *C. violaceum* growth under anaerobic conditions. The indicated strains of *C. violaceum* were diluted to OD_600_ = 0.1 in LB medium or LB plus 0.5% NaNO_3_. The cultures were grown without agitation in an anaerobic jar and the OD_600_ was measured after 48 h. Statistical analysis was performed by Two-way ANOVA and *P* value < 0.05 was considered significant. *P* value < 0.05 = *; *P* value < 0.01 = **; *P* value < 0.001 = ***; *P* value < 0.0001 = ****

Based on genome prediction [5, 6] and experimental data [40], *C. violaceum* can undergo anaerobic respiration using nitrate as an electron acceptor. Considering the remarkable role of OsbR on repressing the *nar* genes, we checked the involvement of *osbR* in anaerobic respiration, growing the *C. violaceum* strains in LB medium in an anaerobic jar (Fig. 7). No bacterial growth occurred in LB unless this medium was supplemented with NaNO_3_, confirming that *C. violaceum* uses nitrate to obtain energy in anaerobic conditions. When compared with the WT strain, the ∆*osbR* strain had an impaired growth, while the WT(*osbR*) strain showed an improved growth (Fig. 7). These data indicate that the overexpression of the *nar* genes (in ∆*osbR*) was detrimental while their repression (in WT(*osbR*)) was beneficial under these conditions. Although *C. violaceum* possesses the enzymes nitrate reductase (Nar), nitrite reductase (Nir), and nitric oxide reductase (Nor) allowing denitrification until nitrous oxide (N_2_O) [5, 6, 40], OsbR affected the expression only of the *nar* genes. Therefore, the elevated expression of the *nar* genes could enhance the reduction of nitrate to nitrite in the *osbR* mutant, causing nitrite accumulation and toxicity. Nitrite accumulation is a challenge in bacteria that lack nitrite reductase [41, 42]. Otherwise, in *P. aeruginosa*, a strain overexpressing the response regulator AtvR had upregulation of all denitrification genes (*nar, nir, nor*, and *nos* operons) and improved growth coupled to anaerobic nitrate reduction [43].

### OsbR represses biofilm formation in *C. violaceum*

**Fig. 8:**
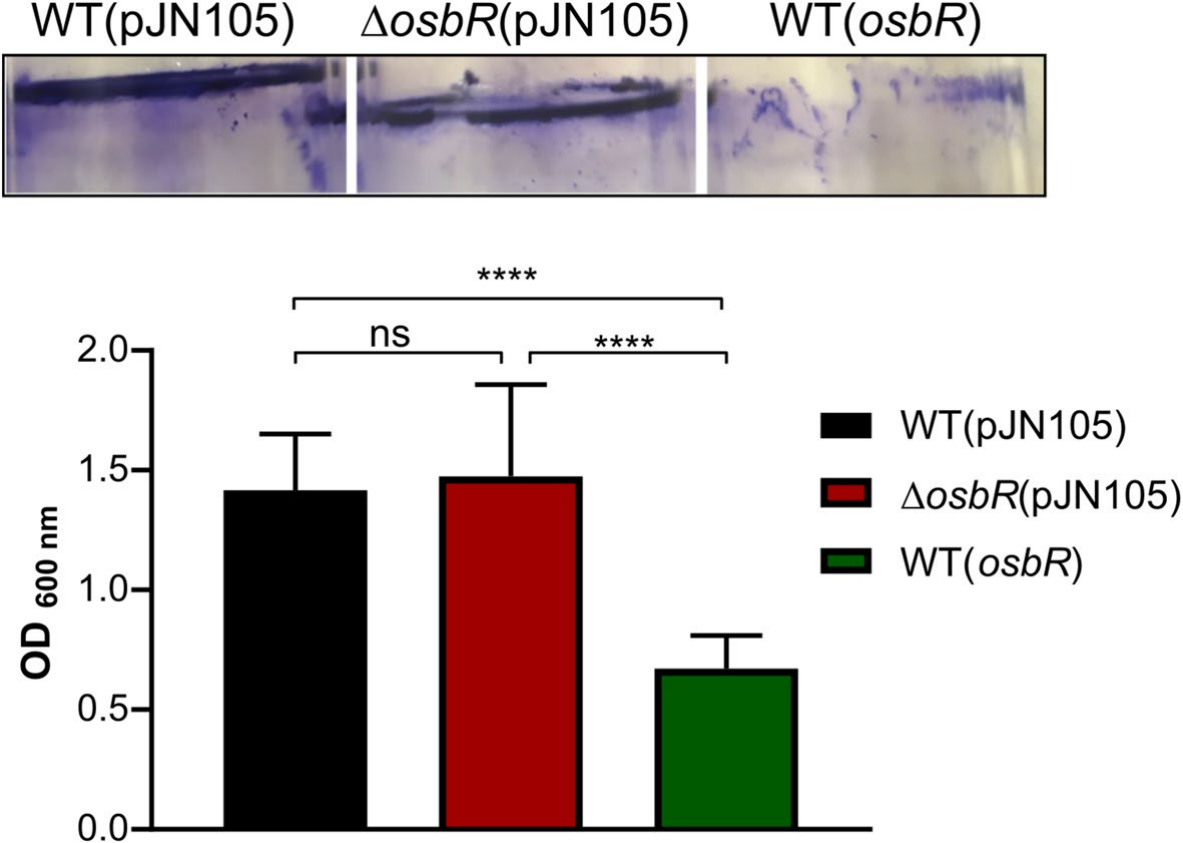
OsbR represses biofilm formation in *C. violaceum.* Strains were diluted to OD_600_ = 0.01 in LB medium, and the biofilm formation was measured after growth in static condition for 24 h. Upper: Representative test tubes for each strain after biofilm staining with violet crystal. Bottom: Quantification of biofilm formation by measurement of OD_600_. Statistical analysis was performed with One-way ANOVA with *P* value < 0.05 being considered significant. ****, *P* value < 0.0001

Several MarR-type regulators homologs to OspR/MgrA have been associated with biofilm formation, including MgrA and SarZ of *S. aureus* [21, 44, 45], AbfR of *S. epidermidis* [23], and AsrR of *E. faecium* [26]. To investigate whether OsbR affects biofilm formation in *C. violaceum*, we performed a biofilm assay with cultures grown statically in LB medium. Deletion of *osbR* had no effect, while overexpression of *osbR* caused a pronounced reduction in the biofilm formation (Fig. 8). Detailed inspection of our microarray data indicated that genes from two clusters encoding putative type I secretion systems and related RTX adhesins (CV_1734-35-36-37 and CV_0513-15-16) were upregulated in the absence of OsbR when comparing WT(*osbR*) with ∆*osbR*(pJN105) (Additional file 2, Table S2). As adhesins are important to surface attachment and biofilm development [46], we propose that the *osbR* overexpression caused a reduction in the biofilm formation owing to the OsbR-mediated repression of these two gene clusters. However, more work will be needed to prove this assumption and to define the role of RTX proteins in *C. violaceum*. In agreement with our data, in *S. aureus* and *E. faecium*, the regulators MgrA and AsrR negatively regulate biofilm production by repressing several surface adhesins [21, 26, 44].

It will be of interest in future studies to address whether OsbR senses oxidants via its cysteine residues to regulate its target genes. Moreover, genetic epistasis analyses could provide a link of the phenotypes related to OsbR (oxidative stress, biofilm formation, and anaerobic growth) and the specific genes of the OsbR regulon.

## Conclusions

In this work, we presented transcriptome and phenotypic characterization of OsbR, a yet unstudied MarR family transcriptional regulator of *C. violaceum*. We showed that OsbR regulates many genes involved in diverse cellular functions as its counterparts OspR/MgrA. Among the OsbR-regulated genes, we highlighted those encoding proteins associated with organic hydroperoxide detoxification (*ohrA/ohrR*), nitrate assimilation (*narK1K2*), anaerobic nitrate respiration (*narGHJI*), and biofilm formation (putative type I secretion systems and associated adhesins). Phenotypic assays using *C. violaceum* strains harboring null mutation or overexpression of *osbR* confirmed the importance of OsbR to organic hydroperoxide resistance (probably by OsbR activation of *ohrA*), anaerobe nitrate respiration (by strong repression of *nar* operons), and biofilm formation (by repression of adhesins). The EMSA assays revealed that reduced but not oxidized OsbR binds to the promoter regions of either repressed or activated genes. Together, the results outline the global function and importance of OsbR as a novel MarR-type regulator in *C. violaceum*.

## Methods

### Bacterial strains and growth conditions

All the bacterial strains and plasmids used in this work are listed in Table 1. The *Chromobacterium violaceum* wild-type (WT) strain ATCC 12472 and its derivative strains were grown at 37 °C in Luria-Bertani (LB) medium supplemented with gentamycin (40 μg/ml) or kanamycin (50 μg/ml), when necessary. Mutant strains harboring an in-frame deletion of *osbR* (Δ*osbR*) and of *osbR* and *ohrR* (Δ*osbR*Δ*ohrR*) were constructed by homologous recombination, using a previously described allelic exchange protocol based on sucrose selection [29, 31–33]. All mutant strains were confirmed by PCR and DNA sequencing (data not shown). To obtain an *osbR* overexpressing strain (WT(*osbR*)), the *osbR* gene with its own promoter was cloned into the pJN105 plasmid, and the resulting construct was introduced in the WT strain by conjugation. DNA cloning was performed by digestion with restriction enzymes of PCR-amplified DNA products (Table 2).

**Table 1:** Bacterial strains and plasmids

Strain or plasmid	Description	Reference or source
*Escherichia coli* strains		
DH5α	Strain for cloning	[47]
S17–1	Strain for plasmid mobilization	[48]
BL21(DE3)	Strain for protein expression	Novagen
*Chromobacterium violaceum* strains		
ATCC 12472	Wild-type strain (Sequenced genome)	[6]
JF3905	Wild-type strain with the CV_3905 gene deleted (∆*osbR*)	This work
JF0210	Wild-type strain with the CV_0210 gene deleted (∆*ohrR*)	[32]
JF39050210	Wild-type strain with the CV_3905 and CV_0210 genes deleted (Δ*osbR*Δ*ohrR*)	This work
WT(pJN105)	Wild-type strain containing the vector pJN105 empty	This work
WT(*osbR*)	Wild-type strain overexpressing *osbR*	This work
∆*osbR*(pJN105)	Mutant *osbR* containing the vector pJN105 empty	This work
∆*osbR*(*osbR*)	Mutant *osbR* strain overexpressing *osbR*	This work
Plasmids		
pNPTS138	Suicide vector containing *oriT sacB*; Kanamycin resistance	D. Alley
pJN105	Broad-host-range vector; Gentamycin resistance	[49]
pET15b	His-tagged protein expression vector; Ampicillin resistance	Novagen
pNPTS138 ∆*osbR*	In frame null deletion of *osbR*	This work
pNPTS138 ∆*ohrR*	In frame null deletion of *ohrR*	[32]
pJN105 *osbR*	Overexpression of *osbR*	This work
pET15b *osbR*	Heterologous expression of the His-OsbR protein	This work

**Table 2:** Primers used in this work

Primer	Sequence (5′ - > 3′)
Mutant and overexpressing strains	
CV3905-del1	TAGGGCCCGAAACTGCGGCTCGAACGCC
CV3905-del2	ATCAAGCTTCATCGAAAGAGTCCCCGTGC
CV3905-del3	ATCAAGCTTGAAAACACGCCGGCCTGAGC
CV3905-del4	TTGGATCCCTGGTGGCGGGCTTTGTTGC
CV_3905-Comp-Fw	GGTACCCTGCAGAGCCAGTCCGCATTAAGGCG
CV_3905-Sup-Rv	GGTTACGAGCTCGACGCTCAGCGTCAGAGCTG
Northern blot	
CV0209-NB-Fw	TGCAGGTGAAGTTGAGCACCCC
CV0209-NB-Rv	CCGATGAAGCAGGCGGAATAGCC
NB CV2534 -Fw	GATGGTGCTGATCGCGATGG
NB CV2534 -Rv	CCAGGGTCTCGATCAGCATC
NB CV2541-Fw	CCATTCTGTCCGCGCTGTTG
NB CV2541-Rv	GCATTTCGTCCATGTCGCGG
NB CV3659-Fw	CACCTCTGGTGCAGGCTTTC
NB CV3659-Rv	GACAGCGACTGGGTGAACTG
NB CV0568-Fw	ACGCCAAGCTGGGACTCAAC
NB CV0568-Rv	GCTCTACCTCCCGTTCGATG
EMSA	
pCV0209-Fw	TTGGATCCTGCCGGAGCGGAATGCAACT
pCV0209-Rv	ATTACTGCAGGCGGTGGCTTCGGCGGTATAC
pCV3905-Fw	GGTACCCTGCAGAGCCAGTCCGCATTAAGGCG
pCV3905-Rv	ATCAAGCTTCATCGAAAGAGTCCCCGT
pCV0210-Fw	CTCGCTGGCCGATCTGGGAC
pCV0210-Rv	GGCGGAATACAGCGCGAAGC
pCV1486Fw	GACCAAGTGATAGACGCCGG
CV1486-DEL2	GGCCTAAAGCTTCTCGGTCATGAGGATGTCTG
pCV2087Fw	CCTCGTAAAAGCGGATGGCG
pCV2087Rv	CGTCGAAACCCTTCATCGCG
pCV2534Fw	GATCACCGACACCCTCAAGC
pCV2534Rv	GCGACAACACCAGCAGCTTG
pCV2545Fw	TGACTCTCCCCCATTGGGAG
pCV2545Rv	ATCGGGATGCCCAGCACCGC
pCV3659Fw	GACCGACAATGACGGCTACG
pCV3659Rv	GCCGAAATGCAGCACGAAAG
CV0208 Fw	GACCATACTAGACAGCTACGCC
CV0208Rv	CAGCGTGGTGATTTGCGGATAG

Underlined letters indicate restriction enzyme recognition sites used for cloning purposes.

### Growth curves

Growth curves of the *C. violaceum* strains were performed in LB medium at 37 °C and 250 rpm agitation in Erlenmeyer flasks. Overnight liquid cultures were diluted to an optical density at 600 nm (OD_600_) of 0.01. Samples were collected at the indicated time points to measure the OD_600_ in a BioPhotometer (Eppendorf). The growth curves under oxidant conditions were performed in 96-well plates. We added 120 μM *tert*-butyl hydroperoxide (TBHP) or 55 μM cumene hydroperoxide (CHP) to the *C. violaceum* LB cultures at OD_600_ of 0.01. The growth was determined by measuring for 24 h the OD_600_ at 15 min intervals in a SpectraMax i3 MiniMax Imaging Cytometer. Data were plotted in one-hour intervals for clarity.

### Growth under anaerobic conditions

Overnight cultures of *C. violaceum* were diluted in 2 mL of LB to OD_600nm_ of 0.1. Cultures were grown in LB or LB plus 5% NaNO_3_ without agitation at 37 °C in an anaerobic jar with a GasPak EZ Anaerobe Container System (catalog number 260001; Becton Dickinson) to establish anaerobic conditions. The growth was determined by measuring the OD_600_ after cultivation by 48 h.

### Biofilm formation

Biofilm was quantified using a crystal violet method as previously described [29]. Briefly, *C. violaceum* liquid cultures at an OD_600_ of 0.01 were grown without agitation at 37 °C for 24 h in 1.5 mL LB in glass tubes. The biofilm was stained with crystal violet, solubilized with ethanol, and quantified by measuring the OD_600_.

### RNA isolation

The *C. violaceum* strains in three biological replicates (for DNA microarray assays) were grown in LB medium at 37 °C and 250 rpm agitation until mid-log phase (OD_600_ of 0.8 to 1.2). The cultures were centrifugated, and the total RNA was extracted with the TRIzol reagent (Ambion) and purified with the illustra RNAspin Mini RNA isolation kit (GE Healthcare). The RNA integrity was evaluated by formaldehyde agarose gel. The absence of DNA contamination was confirmed by PCR. Quantification of total RNA was measured with a NanoDrop spectrophotometer (Thermo Scientific).

### DNA microarray

The procedures for cRNA labeling, hybridization, and washing were performed according to the Agilent Two-Color Microarray-Based Exon Analysis protocol (Agilent Technologies). Briefly, 100 ng RNA of each sample were converted in cRNA and labeled with Cy3 and Cy5 using the Low Input Quick Amp WT Two-Color kit (Agilent Technologies). Equimolar amounts of oppositely labeled cRNA were hybridized at 65 °C by 17 h in a custom-designed oligonucleotide microarray slide [30]. The slides were scanned with an Agilent High-Resolution C Scanner. The data were extracted and normalized by using Agilent Feature Extraction Image Analysis Software (Version 10.7.3). Data analysis to determine the differentially expressed genes was performed as previously described [30, 31]. Briefly, the values for the relative expression of each gene were calculated as the average of the values of all probes corresponding to the same gene (ratio values of at least 27 probes, 9 from each biological replicate). Statistical significance was calculated according to the chi square *p*-value compositional method [50], as implemented in [51]. Briefly, the log-ratio *p*-values for each probe were obtained from Agilent Feature Extraction Software. The global significance level was controlled at 0.05 using the highly stringent Bonferroni multiple correction method. Considering that multiple hypothesis testing were performed (45,220*3 = 135,660, number of probes in biological triplicates), the effective cutoff is 0.05/135660 = 3.68*10^− 7^. Genes were considered differentially expressed if showed simultaneously (i) 2-fold change and (ii) statistical significance at 0.05 (< 3.68*10^− 7^).

### Northern blot

For the microarray validation, we used 7.5 μg of the same RNA samples. For the expression of *ohrA* under oxidant condition, we used 5 μg of total RNA extracted from the WT, Δ*osbR,* Δ*ohrR*, and Δ*osbR*Δ*ohrR* strains grown in LB untreated or treated with 100 μM of cumene hydroperoxide (CHP) for 10 min. After running in denaturing formaldehyde agarose gel, the RNA samples were transferred onto nylon Hybond-XL membranes (GE Healthcare). Specific probes for the indicated genes were generated by PCR (Table 2) and radiolabeled with [α ^32^P]-dCTP according to the DECAprime kit (Ambion) protocol. RNA transfer, membrane hybridization and washing, and signal detection were performed as described [30, 32]. Briefly, RNA was transferred to membranes by capillarity. Membranes were prehybridized and incubated with the labeled probes in 10 ml of ULTRAhyb (Ambion). Washing was performed according to the manufacturer’s instructions and as previously described [30].

### Expression and purification of his-OsbR

The *osbR* gene (CV_3905) was PCR-amplified and cloned into the expression vector pET15b (Table 2). The *E. coli* BL21(DE3) harboring the plasmid pET15b-*osbR* was used to express the recombinant His-tagged OsbR protein after induction with 1 mM IPTG for 2 h at 37 °C. The His-OsbR protein was purified by affinity chromatography in a Ni-NTA superflow column (Qiagen) as previously described [32].

### OsbR dimerization in vitro

The purified His-OsbR protein was incubated with the reductant agent dithiothreitol (DTT) or the oxidants cumene hydroperoxide (CHP), *tert*-butyl hydroperoxide (TBHP), and hydrogen peroxide (H_2_O_2_) at concentrations of 0.1 and 1 mM for 30 min at room temperature. The samples were alkylated with 100 mM N-ethylmaleimide (NEM) for 2 h. The presence of OsbR as monomers or dimers was analyzed after non-reducing SDS-PAGE and Coomassie brilliant blue staining.

### Electrophoretic mobility shift assay (EMSA)

DNA probes corresponding to the promoter region of the indicated genes and the coding region of CV_0208 were amplified by PCR with specific primers (Table 2). These fragments were labeled with [γ^32^P]-ATP by T4 polynucleotide kinase (Thermo Scientific) and purified with the Wizard SV gel and PCR clean-up system (Promega). The interaction of reduced OsbR with the DNA probes was performed as already described [30, 32]. The EMSA with oxidized OsbR was performed by pretreatment of OsbR with 1 mM CHP by 1 h at room temperature.

### Western blot

The *C. violaceum* strains were grown in LB until the mid-log phase. Samples were collected, centrifugated and the pellets were resuspended in an SDS sample buffer. Proteins were resolved by 15% SDS-PAGE and transferred onto a nitrocellulose membrane. The western blot was performed with the Protein Detector LumiGLO Western blot (KPL) kit, using an anti-OsbR polyclonal antiserum (1:1000) developed in mice by a subcutaneous injection of the His-OsbR protein. The six-week-old female BALB/c mice (*n* = 5) used in this study were obtained from the facility Biotério Geral (PUSP-RP), and maintained in the Animal Facilities of the Faculdade de Medicina de Ribeirão Preto (FMRP-USP). The animals were euthanized with an excessive dose of anesthetic (one dose of 200 mg/kg ketamine and 20 mg/kg xylazine).

## Supplementary Information

**Additional file 1: Figure S1** OsbR alignment. **Figure S2** His-OsbR purification by affinity chromatography.

**Additional file 2: Table S1** Comparison of the transcriptome profiles of WT with Δ*osbR.* **Table S2** Comparison of the transcriptome profiles of WT(*osbR*) versus Δ*osbR*(pJN105). **Table S3** List of genes shared among our microarray analyses and the CHP stimulon.

**Additional file 3: Figure S3** Uncropped blot. **Figure S4** Uncropped Northen blot. **Figure S5** Uncropped Northern blot.

## Data Availability

The data supporting the conclusions of this article are included within the article and its additional files. The microarray datasets are available in the Gene Expression Omnibus (GEO) database (https://www.ncbi.nlm.nih.gov/geo/) with the accession number GSE171860.
