# Supplementary material for: The MarR family regulator OsbR controls oxidative stress response, anaerobic nitrate respiration, and biofilm formation in Chromobacterium violaceum
Source: BMC Microbiol. 2021 Nov 4;21:304. doi: 10.1186/s12866-021-02369-x (PMC8567585; doi:10.1186/s12866-021-02369-x)
Supplement: Supplementary file 1 — Additional file 1: Figure S1 OsbR alignment. Figure S2 His-OsbR purification by affinity chromatography. [file 12866_2021_2369_MOESM1_ESM.pdf]

The MarR family regulator OsbR controls oxidative stress response, anaerobic nitrate respiration, and biofilm formation in *Chromobacterium violaceum*

Additional File 1. Figures S1-S2.

A

```
OsbR                                 MKKSDQASFLPVVRELSRTYQAFEQFASYNIRRLGLTPPQFDVVTTLGNSQGMNCKELSD   60
EEG07003.1 (P.ferrooxidans)          -----KTDVLPVIRELVRAFQAFEQYSARHVRELGLTPPQFDVIATLGNTPGMSCKELSE   55
KJV29229.1 (A. magnusonii)           MPHANKELSLSVIRELARTYQAFEQLSAFHIRQMDLTPPQFDVVATLGNTPGMSCKELSE   60
WP_054286986.1 (G. mobilis)          -----EAPFLPVVRELARTFQAFEHFSARHVRQMGLTPPQFDVIATLGNTPGMSCKQLSE   55
TDR78472.1 (P.purpuratum)            MSQSPREPFLPVIRLLAQTYQAFEQFSAFHVRQMGLTPPQFDVVATLGNTRGMSCKELSE   60
RXZ44812.1 (C. cavernae)             MPTATREPFLPVIRELVRTYQAFESFSNAHVRELGLTPPQFDVLATLGNTPGMSCKELSE   60
WP_028497861.1 (M. aerodenitrificans) MTATPAHRFLPVIRELARTYQVFEQLSGNHIRELGLTPPQFDVIATLGNTSGMSCKLLSE   60
RTK97832.1 (N. bacterium)            --------FLPVVRELVRSYQAFELVSARHIRELGLTPPQFDIIATLGNTPGMNCKELGE   52
WP_147694480.1 (V. mureinivorans)    ---------IPLVRELVRTYQAFEQLSSQRIRQHGLTHPQFDVIATLGNTPGMSCRELSE   51
                                              : ::* * :::*.**  :  .:*. .** ****:::****: **.*: *.:

OsbR                                 HTLITKGTLTGVLDRLEDKGIVTRSMQPNDRRSVFVALTPYGQQLFNDAFPAHLDYMHNA   120
EEG07003.1 (P.ferrooxidans)          KTLITKGTLTGVIDRLAEKGVVTRQERPEDRRSVFISLTAAGEELFRQVFPAHCDYMHQA   115
KJV29229.1 (A. magnusonii)           KTLITKGTLTGVIDRLIDKGIVTRCTLEQDRRSVMVALTAQGEALFQQVFPAHLAFMKPA   120
WP_054286986.1 (G. mobilis)          KTLITKGTLTGVIDRLEEKGLVTRTPLPQDRRSIFIALTAAGEALFQQVFPAHLAYMQAA   115
TDR78472.1 (P.purpuratum)            KTLITKGTLTGVVDRLCDKGIVQRTTMEHDRRSVFVALTARGEALFEEAFPAHLMHMRQA   120
RXZ44812.1 (C. cavernae)             QTLITKGTLTGVIDRLVDKGLVSRTAQEEDRRSVLIALTDTGQALFDQIFPAHLAHMRQA   120
WP_028497861.1 (M. aerodenitrificans) KTLITKGTLTGVVDRLIDKGLVSRRIPDEDRRSVFIALTTEGEALFARVFPAHLDHMQKL   120
RTK97832.1 (N. bacterium)            KTLITKGTLTGVLDRLTDKGLICRSTPPEDRRSIFICLTPAGETLFRQAFPAHLQFLDNI   112
WP_147694480.1 (V. mureinivorans)    RTLITKGTLTGVLDRLLDKGLITRTVPEHDRRSLFVALTPAGEAVFDSAFPDVVSHCGEA   111
                                     :***********:*** :**:: *    .****:::.**  *: :*   **    .

OsbR                                 FQQFGEQDLSGFCNELGRLRASFSQALEQQENTPA  155
EEG07003.1 (P.ferrooxidans)          FSQCSRDQLELWSNQLAGLKTVLNQALEKQEN---  147    59.18%
KJV29229.1 (A. magnusonii)           FDLLEPADMTQLCQQLARLRAAFNQVLEQKKN---  152    59.21%
WP_054286986.1 (G. mobilis)          FDGFDQTQLDAFCTALTNLRSAF------------  138    61.59%
TDR78472.1 (P.purpuratum)            FTAFDSKDYDLCATELKRLRDAFTVAMAE------  149    59.73%
RXZ44812.1 (C. cavernae)             FAGLDNDDFSRICAELAKLRRSIGLAME-------  148    59.46%
WP_028497861.1 (M. aerodenitrificans) FDTFSETELEQIQHMLARLRTGLDTA---------  146    57.53%
RTK97832.1 (N. bacterium)            FGRLPPATLDHLQTSLQALRQAFEQEIK-------  140    58.57%
WP_147694480.1 (V. mureinivorans)    FAHLDDASLNQHIAMLAALRAAIGQKLEPAE----  142    56.34%
                                     *               *  *:  :
```

B

```
OsbR                 ----------------MKKSDQASF--LPVVRELSRTYQAFEQFASYNIRRLGLTPPQFD    42
OspR WP_003114767.1  MSTRGKVARPDGVEESLLLDNQLCFKLYAASRAVIRG------YRP-LLEQIGLTYPQYL    53
BmoR WP_005784476.1  -------------------MNTIC--------KMRDIYKALSIFETAFEEVYGISLNEAM    33
MosR WP_003405595.1  ------------MGKGAAFDECACYTTRRAARQLGQA------YDR-ALRPSGLTNTQFS    41
AbfR WP_002467790.1  -----------MKQEQMRLANQLCFSAYNVSRLFAQF------YEK-KLKQFGITYSQYL    42
MgrA WP_001283444.1  ------------MSDQHNLKEQLCFSLYNAQRQVNRY------YSNKVFKKYNLTYPQFL    42
                                            :  .         .         :     .  .::  :

OsbR                 VVTTLGNSQG-----MNCKELSDHTLITKGTLTGVLDRLEDKGIVTRSMQPNDRRSVFVA    97
OspR WP_003114767.1  VMLVLWEWHASPPEQPTVKALGDRLLLDSGTLTPLLKRLEQLGLVDRRRASHDEREVHLT    113
BmoR WP_005784476.1  VLCALRE----AGKEITSTAIAERTEMAPSHTSKVIRAVEDKGLIRRALGEVDKRQMYFS    89
MosR WP_003405595.1  TLAVISLSEGSAGIDLTMSELAARIGVERTTLTRNLEVMRRDGLV-RVMAGADARCKRIE    100
AbfR WP_002467790.1  VLLTLWEENP-----QTLNSIGRHLDLSSNTLTPLLKRLEQSGWVKRERQQSDKRQLIIT    97
MgrA WP_001283444.1  VLTILWDESP-----VNVKKVVTELALDTGTVSPLLKRMEQVDLIKRERSEVDQREVFIH    97
                     .:  :          . . :  .  :     :  :  :.  . : *     * *   .

OsbR                 LTPYGQQLFNDAFPAHLDYMHNAFQQFGEQD-----LSGFCNELGRLRASFSQALEQQEN    152
OspR WP_003114767.1  LTVPGITLRERVIPLRQQLICSTGFDLNEMF-------DLHQRLGGLLSRFRLVVGG---    163
BmoR WP_005784476.1  LTEAGKKRLNELDLDK-----------VEIP-------EM---LKPLI------------    116
MosR WP_003405595.1  LTAKGRAALQKAVPLWRGVQAEVTASVGDWPRVRRDIANL----GQAAEACR--------    148
AbfR WP_002467790.1  LTDNGQQQQEAVFEAISSCL-PQEFDTTEYDETKYVFEELEQTLKHLIEK----------    146
MgrA WP_001283444.1  LTDKSETIRPELSNASDKVASASSLSQDEVK-------ELNRLLGKVIHAFDETKEK---    147
                     **  .                         :          :

OsbR                 TPA  155
OspR WP_003114767.1  ---  163   34.88%
BmoR WP_005784476.1  ---  116   25.49%
MosR WP_003405595.1  ---  148   26.73%
AbfR WP_002467790.1  ---  146   33.33%
MgrA WP_001283444.1  ---  147   30.43%
```

Fig. S1 OsbR alignment. a. Clustal Omega alignment of *C. violaceum* OsbR with highly similar MarR family transcriptional regulators. Proteins of *Pseudogulbenkiania ferrooxidans* (EEG07003.1), *Aquitalea magnusonii* (KJV29229.1), *Gulbenkiania mobilis* (WP_054286986.1), *Paludibacterium purpuratum* (TDR78472.1), *Crenobacter cavernae* (RXZ44812.1), *Microvirgula aerodenitrificans* (WP_028497861.1), *Neisseriaceae bacterium* (RTK97832.1), *Vogesella mureinivorans* (WP_147694480.1) found by BLASTp analysis. High conserved Cys55 and less conserved Cys133 residues are indicated in yellow. Percentages indicate protein identity with OsbR accordingly with NCBI BLAST. **b.** Clustal Omega alignment of OsbR with *Pseudomonas aeruginosa* OspR, *Bacteroides fragilis* BmoR, *Mycobacterium tuberculosis* MosR, *Staphylococcus epidermidis* AbfR, and *Staphylococcus aureus* MgrA. Conserved cysteines are indicated in yellow, as well as both OsbR cysteine residues. Percentages indicate protein identity with OsbR accordingly with NCBI BLAST.

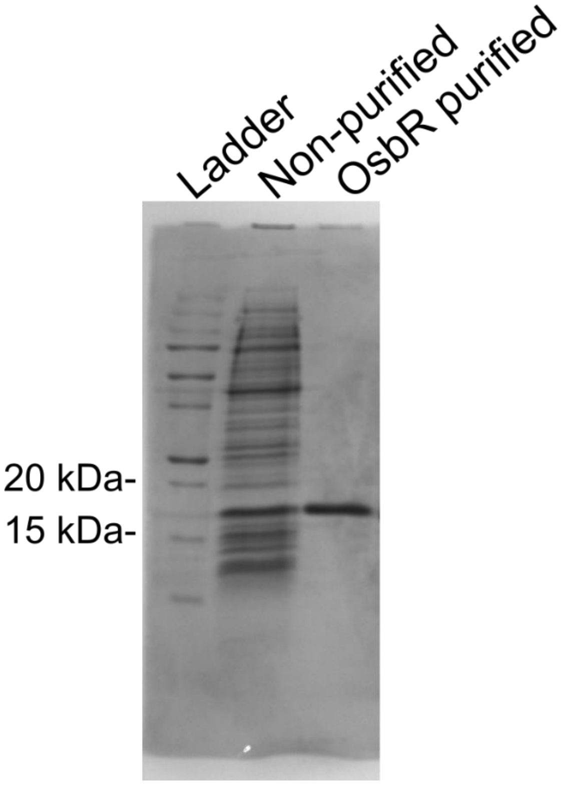

Fig. S2 His-OsbR purification by affinity chromatography. The His-OsbR was overexpressed in *E. coli* BL21(DE3) with IPTG (Non-purified). The purification of His-OsbR in a Ni-NTA column presented high efficiency and purity (OsbR purified). His-OsbR has approximately 17 kDa.
