# Supplementary material for: The MarR family regulator OsbR controls oxidative stress response, anaerobic nitrate respiration, and biofilm formation in Chromobacterium violaceum
Source: BMC Microbiol. 2021 Nov 4;21:304. doi: 10.1186/s12866-021-02369-x (PMC8567585; doi:10.1186/s12866-021-02369-x)
Supplement: Supplementary file 2 — Additional file 2: Table S1 Comparison of the transcriptome profiles of WT with ΔosbR. Table S2 Comparison of the transcriptome profiles of WT(osbR) versus ΔosbR(pJN105). Table S3 List of genes shared among our microarray analyses and the CHP stimulon. [file 12866_2021_2369_MOESM2_ESM.pdf]

The MarR family regulator OsbR controls oxidative stress response, anaerobic nitrate respiration, and biofilm formation in *Chromobacterium violaceum*

Additional File 3. Figure S3-S5.

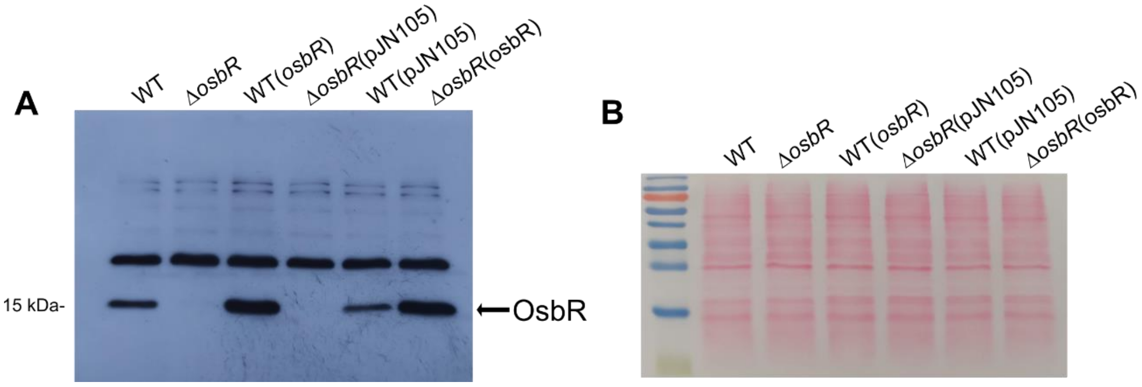

Fig. S3 Uncropped blot. a, b. Full-length blot (a) and Ponceau-stained membrane (b) related to the Fig. 1a.

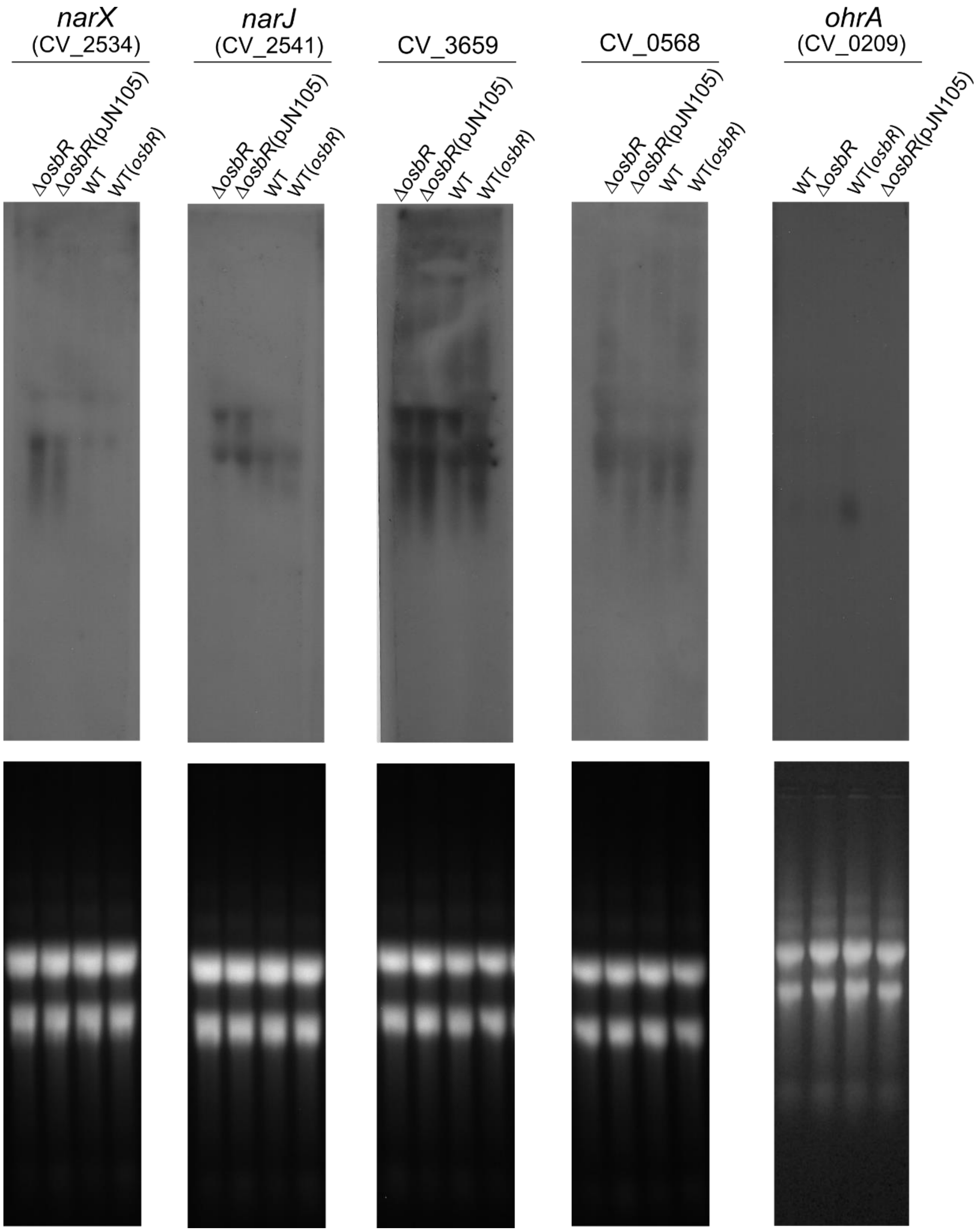

Fig. S4 Uncropped Northen blot. Above, autoradiography of the indicated genes. Below, gel of respective samples showing RNA loading. Related to the Fig. 3.

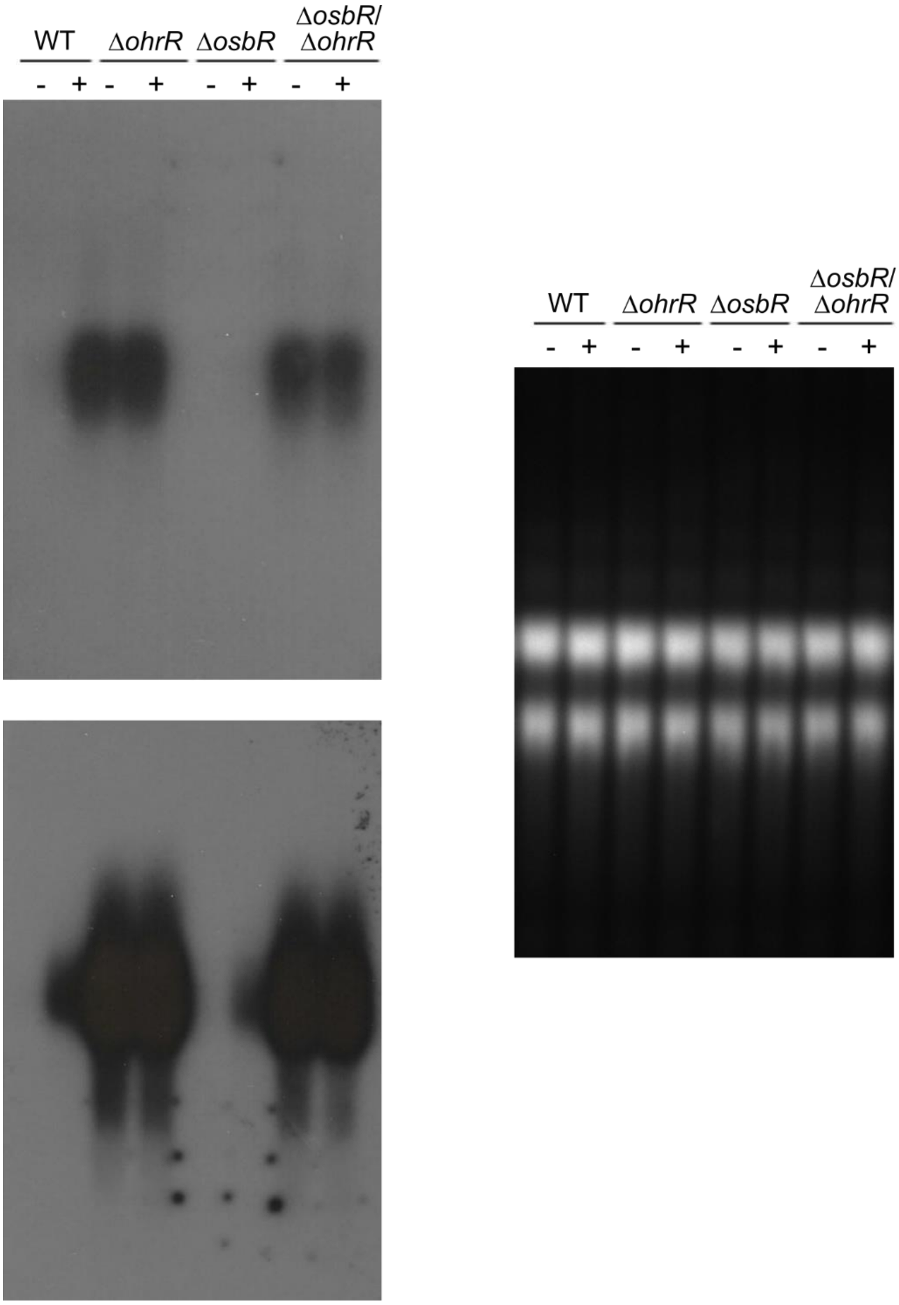

Fig. S5 Uncropped Northern blot. Left**,** autoradiography less (above) or more (below) exposed for *ohrA***.** Right, gel of respective samples showing RNA loading**.** Related to the Fig. 6a.
