# Supplementary material for: The MarR family regulator OsbR controls oxidative stress response, anaerobic nitrate respiration, and biofilm formation in Chromobacterium violaceum
Source: BMC Microbiol. 2021 Nov 4;21:304. doi: 10.1186/s12866-021-02369-x (PMC8567585; doi:10.1186/s12866-021-02369-x)
Supplement: Supplementary file 3 — Additional file 3: Figure S3 Uncropped blot. Figure S4 Uncropped Northen blot. Figure S5 Uncropped Northern blot. [file 12866_2021_2369_MOESM3_ESM.pdf]

The MarR family regulator OsbR controls oxidative stress response, anaerobic nitrate respiration, and biofilm formation in *Chromobacterium violaceum*

Additional File 2. Tables S1, S2 and S3.

Table S1 Comparison of the transcriptome profiles of WT with Δ*osbR*

Gene		Predicted function	Fold change Δ*osbR*/WT	P-value Δ*osbR*/WT
**Genes downregulated in the Δ*osbR***				
**Amino Acid Metabolism**				
CV_0568 *		Anthranilate synthase	0.178	$< 10^{-16}$
CV_2803 *		Probable peptide synthetase protein	0.287	$< 10^{-16}$
CV_2804 *		Diaminobutyrate-2-oxoglutarate transaminase	0.344	$< 10^{-16}$
CV_3429 *		Glycine dehydrogenase	0.438	$< 10^{-16}$
CV_3431 *		Aminomethyltransferase / glycine cleavage system T protein	0.446	$< 10^{-16}$
**Fatty Acid Metabolism**				
CV_1541 *		3-oxoacyl-[acyl-carrier-protein] synthase II	0.12	$< 10^{-16}$
CV_1546 *		3-oxoacyl-[acyl-carrier protein] reductase	0.403	$< 10^{-16}$
**Efflux systems/Transport**				
CV_1219 *		Permease transmembrane protein	0.381	$< 10^{-16}$
CV_1989		Probable porin protein	0.466	$< 10^{-16}$
CV_1629		Glycine betaine/proline transport system substrate-binding protein	0.443	$< 10^{-16}$
CV_2248 *	*fucP*	MFS transporter, FHS family, L-fucose permease	0.269	$< 10^{-16}$
**Iron Metabolism**				
CV_1547 *		Ferredoxin	0.379	$< 10^{-16}$
**Chemotaxis**				
CV_3285 *		Methyl-accepting chemotaxis protein	0.4	$< 10^{-16}$
**Metabolic pathways**				
CV_2247 *		Transaldolase	0.346	$< 10^{-16}$
**Signal Transduction**				
CV_2513		Probable sensor histidine kinase/response regulator	0.486	$< 10^{-16}$
**MarR transcriptional Regulator**				
CV_3905 *		Probable transcriptional regulator/ MarR family	0.021	$< 10^{-16}$
**Hypothetical Proteins**				
67196_2279		Hypothetical protein	0.484	$6.7\ 10^{-16}$
67196_4197		Hypothetical protein	0.387	$3.4\ 10^{-10}$
67196_4249		Hypothetical protein	0.439	$< 10^{-16}$
CV_0071		Hypothetical protein	0.343	$7.8\ 10^{-15}$

CV_0123		Hypothetical protein	0.359	$< 10^{-16}$
CV_0285		Hypothetical protein	0.464	$< 10^{-16}$
CV_0512		Hypothetical protein	0.42	$< 10^{-16}$
CV_0567 *		Hypothetical protein	0.26	$< 10^{-16}$
CV_0569 *		Hypothetical protein	0.159	$< 10^{-16}$
CV_0570 *		Hypothetical protein	0.154	$< 10^{-16}$
CV_0571 *		Hypothetical protein	0.426	$< 10^{-16}$
CV_0572 *		Uncharacterized protein	0.321	$< 10^{-16}$
CV_0592 *		Conserved hypothetical protein	0.33	$< 10^{-16}$
CV_0636		Hypothetical protein	0.459	$< 10^{-16}$
CV_1218 *		Hypothetical protein	0.302	$1.7\ 10^{-8}$
CV_1527		Hypothetical protein	0.385	$< 10^{-16}$
CV_1540 *		Hypothetical protein	0.31	$< 10^{-16}$
CV_1543 *		Hypothetical protein	0.164	$< 10^{-16}$
CV_1544 *		Hypothetical protein	0.116	$< 10^{-16}$
CV_1545 *		Conserved hypothetical protein	0.209	$< 10^{-16}$
CV_2246 *		Hypothetical protein	0.32	$< 10^{-16}$
CV_2249 *		Hypothetical protein	0.291	$< 10^{-16}$
CV_2269		Hypothetical protein	0.442	$< 10^{-16}$
CV_2807		Hypothetical protein	0.393	$< 10^{-16}$
CV_3142 *		Hypothetical protein	0.319	$< 10^{-16}$
CV_3234		Conserved hypothetical protein	0.48	$< 10^{-16}$
CV_4224 *		Conserved hypothetical protein	0.219	$< 10^{-16}$
**Genes upregulated in the Δ*osbR***				
**Transmembrane Transport**				
CV_1800		Probable permease	2.227	$7.5\ 10^{-10}$
**Metabolic Pathways**				
CV_2532 *	*moaA2*	Molybdenum cofactor biosynthesis protein	2.362	$< 10^{-16}$
**Nitrogen metabolism**				
CV_2534 *	*narX*	Transmembrane nitrate/nitrite sensor kinase transcription regulator protein	2.494	$< 10^{-16}$
CV_2540 *	*narI*	Nitrate reductase gamma subunit	5.439	$< 10^{-16}$
CV_2541 *	*narJ*	Nitrate reductase delta subunit	4.73	$< 10^{-16}$
CV_2542 *	*narH*	Nitrate reductase / nitrite oxidoreductase, beta subunit	4.823	$< 10^{-16}$
CV_2543 *	*narG*	Respiratory nitrate reductase alpha chain	4.973	$< 10^{-16}$
CV_2544 *	*narK2*	Nitrite extrusion protein	6.301	$< 10^{-16}$
CV_2545 *	*narK1*	Nitrite extrusion protein	4.952	$< 10^{-16}$
**RNA binding protein**				
CV_3799		RNA-binding protein/ conserved hypothetical protein	2.531	$< 10^{-16}$
**Efflux systems**				
CV_0993		MFS transporter, AAHS family, 4-hydroxybenzoate transporter	3.041	$< 10^{-16}$

CV_2845	Probable efflux transporter	2.273	$< 10^{-16}$
	**Hypothetical Proteins**		
CV_1337	Hypothetical protein	2.577	$< 10^{-16}$
CV_1521	Probable two-component sensor	2.534	$< 10^{-16}$
CV_1639	Conserved hypothetical protein	3.54	$< 10^{-16}$
CV_1848	Conserved hypothetical protein	2.531	$1.6\ 10^{-14}$
CV_2107	Hypothetical protein	4.749	$< 10^{-16}$
CV_2109	Conserved hypothetical protein	2.486	$< 10^{-16}$
CV_2166	Conserved hypothetical protein	2.462	$3.4\ 10^{-15}$
CV_3601	Hypothetical protein	2.82	$< 10^{-16}$
CV_4399	Hypothetical protein	2.557	$1.5\ 10^{-8}$

*Genes shared between our both microarrays

Table S2 Comparison of the transcriptome profiles of WT(*osbR*) versus Δ*osbR*(pJN105)

Open reading frame	Gene	Predicted function	Fold change Δ*osbR*(pJN105)/WT(*osbR*)	P-value Δ*osbR*(pJN105)/WT(*osbR*)
		**Genes downregulated in the Δ*osbR*(pJN105)**		
		**Oxidative Stress**		
CV_0209	*ohrA*	organic hydroperoxide resistance protein	0.17	$< 10^{-16}$
CV_0210	*ohrR*	Probable transcriptional regulator, MarR family	0.35	$< 10^{-16}$
		**Nitrogen Metabolism**		
CV_4002	*glnK*	Nitrogen regulatory protein P-II-2	0.27	$< 10^{-16}$
CV_4003	*amtB*	Ammonium transporter	0.45	$< 10^{-16}$
		**Virulence**		
CV_3143		Probable mannose-binding lectin precursor	0.22	$< 10^{-16}$
		**Efflux Systems**		
CV_1219 *		Probable permease transmembrane protein	0.05	$< 10^{-16}$
CV_2248 *	*fucP*	Fucose permease	0.07	$< 10^{-16}$
CV_4370	*aroP*	Aromatic amino acid transport protein	0.24	$< 10^{-16}$
		**Transcriptional Regulators**		
CV_2087		Probable transcriptional regulator, MerR family	0.13	$< 10^{-16}$
CV_4290		Probable transcriptional regulator LysR-family	0.42	$< 10^{-16}$
		**Chemotaxis**		
CV_3285 *		Methyl-accepting chemotaxis protein	0.37	$< 10^{-16}$
		**Fatty Acid Metabolism**		
CV_1541 *		3-oxoacyl-[acyl-carrier-protein] synthase II	0.09	$< 10^{-16}$

CV_1546 *		3-oxoacyl-[acyl-carrier protein] reductase	0.23	< 10^{-16}
		**Amino Acid Metabolism**		
CV_0568 *		Anthranilate synthase	0.08	< 10^{-16}
CV_1762		3-methylcrotonyl-CoA carboxylase alpha subunit	0.36	< 10^{-16}
CV_1763		Methylglutaconyl-CoA hydratase	0.36	< 10^{-16}
CV_1764		3-methylcrotonyl-CoA carboxylase beta subunit	0.33	< 10^{-16}
CV_1766		isovaleryl-CoA dehydrogenase	0.16	< 10^{-16}
CV_2085	*mmsA1*	Methylmalonate-semialdehyde dehydrogenase	0.29	< 10^{-16}
CV_2088	*atoB*	Acetyl-CoA C-acetyltransferase	0.31	< 10^{-16}
CV_2802		Probable peptide synthetase protein	0.22	< 10^{-16}
CV_2803 *		Probable peptide synthetase protein	0.08	< 10^{-16}
CV_2804 *		Probable diaminobutyrate-pyruvate transaminase	0.08	< 10^{-16}
CV_3429 *	*gcvP*	Glycine cleavage system P protein	0.42	< 10^{-16}
CV_3430	*gcvH*	Glycine cleavage system H protein	0.31	< 10^{-16}
CV_3431 *	*gcvT*	Glycine cleavage system T protein	0.24	< 10^{-16}
		**Metabolic pathways**		
CV_0190		Glyceraldehyde 3-phosphate dehydrogenase	0.42	< 10^{-16}
CV_1222		Probable aldehyde dehydrogenase	0.47	< 10^{-16}
CV_2086		3-hydroxybutyryl-CoA dehydrogenase	0.17	< 10^{-16}
CV_2247 *		Transaldolase	0.08	< 10^{-16}
CV_4289	*proA*	Glutamate-5-semialdehyde dehydrogenase	0.22	< 10^{-16}
		**Iron Metabolism**		
CV_0398	*exbD2*	Biopolymer transport exbD transmembrane protein	0.40	< 10^{-16}
CV_0399		Probable exbB-like biopolymer transport	0.40	< 10^{-16}
CV_0400		Periplasmic protein TonB	0.37	< 10^{-16}
CV_1481	*aroF*	2-dehydro-3-deoxy-phosphoheptonate aldolase	0.41	< 10^{-16}
CV_1482	*cbaA (entA)*	2,3-dihydro-2,3-dihydroxybenzoate dehydrogenase	0.39	< 10^{-16}
CV_1483	*cbaB (entB)*	Isochorismatase	0.47	< 10^{-16}
CV_1484	*cbaE (entE)*	2,3-dihydroxybenzoate-AMP ligase	0.38	< 10^{-16}
CV_1485	*cbaC (entC)*	Isochorismate synthase EntC / MenF	0.39	< 10^{-16}
CV_1486	*cbaF (entF)*	Enterobactin synthetase component F	0.42	< 10^{-16}
CV_1547 *	*fdxA2*	ferredoxin	0.21	< 10^{-16}
		**MarR transcriptional Regulator**		
CV_3905 *		Probable transcriptional regulator/ MarR family	0.01	< 10^{-16}
		**Hypothetical Proteins**		
67196_3489		Hypothetical protein	0.07	< 10^{-16}

67196_3491		Hypothetical protein	0.07	$< 10^{-16}$
CV_0247		Hypothetical protein	0.33	$< 10^{-16}$
CV_0567 *		Hypothetical protein	0.11	$< 10^{-16}$
CV_0569 *		Hypothetical protein	0.08	$< 10^{-16}$
CV_0570 *		Hypothetical protein	0.09	$< 10^{-16}$
CV_0571 *		Hypothetical protein	0.37	$< 10^{-16}$
CV_0572 *		Hypothetical protein	0.11	$< 10^{-16}$
CV_0592 *		Conserved hypothetical protein	0.14	$< 10^{-16}$
CV_1218 *		Hypothetical protein	0.29	$1.1\ 10^{-9}$
CV_1220		Hypothetical protein	0.16	$< 10^{-16}$
CV_1221		Hypothetical protein	0.34	$< 10^{-16}$
CV_1223		Hypothetical protein	0.39	$< 10^{-16}$
CV_1244		Conserved hypothetical protein	0.45	$1.2\ 10^{-11}$
CV_1475		Hypothetical protein	0.36	$< 10^{-16}$
CV_1540 *		Hypothetical protein	0.31	$< 10^{-16}$
CV_1542		Hypothetical protein	0.12	$< 10^{-16}$
CV_1543 *		Hypothetical protein	0.13	$< 10^{-16}$
CV_1544 *		Hypothetical protein	0.06	$< 10^{-16}$
CV_1545 *		Conserved hypothetical protein	0.07	$< 10^{-16}$
CV_1548		Conserved hypothetical protein	0.41	$< 10^{-16}$
CV_1765		Conserved hypothetical protein	0.25	$< 10^{-16}$
CV_2246 *		Hypothetical protein	0.08	$< 10^{-16}$
CV_2249 *		Hypothetical protein	0.05	$< 10^{-16}$
CV_2970		Conserved hypothetical protein	0.34	$< 10^{-16}$
CV_3142 *		Hypothetical protein	0.35	$< 10^{-16}$
CV_3159		Conserved hypothetical protein	0.31	$< 10^{-16}$
CV_3565		Hypothetical protein	0.16	$< 10^{-16}$
CV_3566		Hypothetical protein	0.05	$< 10^{-16}$
CV_4224 *		Conserved hypothetical protein	0.11	$< 10^{-16}$
	**Genes upregulated in the Δ*osbR*(pJN105)**			
		**Nitrogen Metabolism**		
CV_1411		Nitrite transporter NirC	19.13	$< 10^{-16}$
CV_2188	*pphA*	Phosphoprotein phosphatase	9.96	$< 10^{-16}$
CV_2534 *	*narX*	Transmembrane nitrate/nitrite sensor kinase transcription regulator protein	16.12	$< 10^{-16}$
CV_2535	*narL*	Nitrate/nitrite response regulator protein narL	11.10	$< 10^{-16}$
CV_2540 *	*narI*	Nitrate reductase	47.56	$< 10^{-16}$
CV_2541 *	*narJ*	Nitrate reductase	42.05	$< 10^{-16}$
CV_2542 *	*narH*	Nitrate reductase	30.23	$< 10^{-16}$
CV_2543 *	*narG*	Respiratory nitrate reductase alpha chain	21.89	$< 10^{-16}$
CV_2544 *	*narK2*	Nitrite extrusion protein	52.67	$< 10^{-16}$

CV_2545 *	*narK1*	Nitrite extrusion protein	19.53	$< 10^{-16}$
		**Aerobic Respiration**		
CV_1174		Probable cytochrome-c oxidase, subunit I	2.23	$< 10^{-16}$
CV_2449		Probable coenzyme F390 synthetase	5.75	$< 10^{-16}$
CV_3657	*cioB*	Cytochrome bd-I oxidase subunit II	6.95	$< 10^{-16}$
CV_3658	*cioA*	Cytochrome d ubiquinol oxidase subunit I	5.17	$< 10^{-16}$
		**Virulence**		
CV_0304	*rarD*	Chloramphenicol-sensitive protein	5.53	$< 10^{-16}$
CV_0513	*hlyB*	Hemolysin B	2.61	$< 10^{-16}$
CV_0516		Probable calcium binding hemolysin	3.05	$< 10^{-16}$
CV_2419		Probable oxygen-regulated invasion protein; cell invasion protein	3.05	$< 10^{-16}$
CV_2422		Probable peptidyl-dipeptidase Dcp/(GenBank) cell invasion protein - cytoplasmic	2.93	$< 10^{-16}$
CV_2423	*prgH*	Pathogenicity 1 island effector protein	2.80	$< 10^{-16}$
		**Efflux systems**		
CV_0771		MFS transporter, DHA1 family, bicyclomycin/chloramphenicol resistance protein	7.45	$< 10^{-16}$
CV_1105	*gltS*	Sodium/glutamate symport carrier protein	3.99	$< 10^{-16}$
CV_1409	*sdaC*	Serine transporter	4.94	$< 10^{-16}$
CV_1726		Inner membrane transporter RhtA	3.77	$< 10^{-16}$
CV_1734		Outer membrane protein, adhesin transport system	7.29	$< 10^{-16}$
CV_1735	*cydC*	ATP-binding/permease fusion ABC transporter	6.86	$< 10^{-16}$
CV_1736	*hylD*	Membrane fusion protein, adhesin transport system	4.36	$< 10^{-16}$
CV_1737		Conserved hypothetical protein	2.99	$< 10^{-16}$
CV_1901		Probable transport protein	3.29	$< 10^{-16}$
CV_3677		Cobalt-zinc-cadmium efflux system protein	2.36	$< 10^{-16}$
		**Transcriptional Regulators**		
CV_0294	*sir2*	Transcriptional regulator	2.45	$< 10^{-16}$
CV_1451		Probable transcriptional regulator	3.59	$< 10^{-16}$
CV_1664		Two-component system, LytT family, response regulator	10.65	$< 10^{-16}$
CV_1665		Probable two-component system sensor kinase	5.06	$< 10^{-16}$
CV_1905		Probable transcription regulator	5.02	$< 10^{-16}$
CV_3659		Probable transcriptional regulator marR family	4.27	$< 10^{-16}$
		**Chemotaxis**		
CV_1698		Methyl-accepting chemotaxis protein	3.12	$< 10^{-16}$
CV_4244		Probable methyl-accepting chemotaxis transducer	2.74	$< 10^{-16}$

		**Amino Acid Metabolism**		
CV_0118	*gltK*	Glutamate/aspartate transport system permease protein	3.59	$< 10^{-16}$
CV_0119	*gltJ*	Glutamate/aspartate transport system permease	4.18	$< 10^{-16}$
CV_0120		Glutamate/aspartate transport system substrate-binding protein	3.63	$< 10^{-16}$
CV_1408	*sdaA2*	L-serine dehydratase	3.58	$< 10^{-16}$
CV_1914	*dadA2*	D-amino acid dehydrogenase	4.37	$< 10^{-16}$
CV_1915	*alr*	Alanine racemase	5.07	$< 10^{-16}$
CV_1934	*metY*	O-acetylhomoserine (thiol)-lyase	2.84	$< 10^{-16}$
CV_2223		Probable sodium/alanine symporter	3.86	$< 10^{-16}$
CV_2382	*tyrB1*	Aromatic-amino-acid transaminase	2.81	$< 10^{-16}$
CV_3381	*dcp2*	Probable peptidyl-dipeptidase Dcp	2.96	$< 10^{-16}$
CV_3678	*nadA*	Quinolinate synthetase	2.40	$< 10^{-16}$
CV_4084		Putative protease	3.63	$< 10^{-16}$
		**Metabolic Pathways**		
CV_0145	*zwf*	Glucose-6-phosphate 1-dehydrogenase	2.78	$< 10^{-16}$
CV_0515		Probable glycosyltransferase	4.75	$< 10^{-16}$
CV_0979	*ptsG*	Protein-N p-phosphohistidine-sugar phosphotransferase	3.96	$< 10^{-16}$
CV_0980		Probable phosphoenolpyruvate-protein phosphotransferase	4.29	$< 10^{-16}$
CV_1137	*adhE*	Acetaldehyde dehydrogenase	2.87	$< 10^{-16}$
CV_1528		Probable synthetase/amidase	2.65	$< 10^{-16}$
CV_1653	*hemG*	Protoporphyrinogen oxidase	3.28	$< 10^{-16}$
CV_1662	*cstA1*	Carbon starvation protein	34.40	$< 10^{-16}$
CV_1902		Probable hydrolase	2.42	$< 10^{-16}$
CV_2446		3-oxoacyl-[acyl-carrier-protein] synthase III	5.10	$< 10^{-16}$
CV_2447		Probable dehydrogenase	5.64	$< 10^{-16}$
CV_2532 *	*moaA2*	Molybdenum cofactor biosynthesis protein A	17.23	$< 10^{-16}$
CV_2656		Probable cytochrome P450 hydroxylase	2.31	$< 10^{-16}$
CV_2697		Probable tldD protein family	3.69	$< 10^{-16}$
CV_3026	*lldP*	L-lactate permease	3.48	$< 10^{-16}$
CV_3027		Linoleoyl-CoA desaturase/ probable ferredoxin	2.48	$< 10^{-16}$
CV_3054	*fruA*	Protein-N p-phosphohistidine-sugar phosphotransferase	3.22	$< 10^{-16}$
CV_3298	*lamB*	Maltoporin precursor	9.61	$< 10^{-16}$
CV_3299	*treC*	Trehalose-6-phosphate hydrolase	10.59	$< 10^{-16}$
CV_3300	*treB*	Protein-N p-phosphohistidine-sugar phosphotransferase	10.78	$< 10^{-16}$
CV_3304	*aceB*	Malate synthase A	5.24	$< 10^{-16}$
CV_3736		Putative acetyltransferase	2.34	$< 10^{-16}$
		**Secondary metabolites production**		

CV_1682	*hcnC*	Hydrogen cyanide synthase HcnC	5.86	< 10^{-16}
CV_1683	*hcnB*	Hydrogen cyanide synthase HcnB	5.43	< 10^{-16}
CV_1684	*hcnA*	Hydrogen cyanide synthase HcnA	4.59	< 10^{-16}
		**Sulfur metabolism**		
CV_1828	*cysA*	Sulfate transport system ATP-binding protein	2.41	< 10^{-16}
CV_1829	*cysW*	Sulfate transport system permease protein CysW	2.82	< 10^{-16}
CV_1830	*cysU*	Sulfate transport system permease protein	2.79	< 10^{-16}
CV_1832	*sbp*	Sulfate transport system sulfate-binding protein	5.84	< 10^{-16}
CV_1904		O-acetylserine/cysteine efflux transporter	7.35	< 10^{-16}
CV_2855	*tauD*	Taurine dioxygenase	5.37	< 10^{-16}
CV_2856	*tauC*	Taurine transport system permease protein	9.77	< 10^{-16}
CV_2857	*tauB*	Taurine ATP-binding component of a transport system	9.15	< 10^{-16}
CV_2858	*tauA*	Taurine transport system substrate-binding protein	6.59	< 10^{-16}
CV_2981		Cystine transport system ATP-binding protein	2.30	< 10^{-16}
CV_2982		Cystine transport system permease protein	2.54	< 10^{-16}
CV_2983		Cystine transport system substrate-binding protein	2.42	< 10^{-16}
		**Fatty acid metabolism**		
67196_2381		C-5 sterol desaturase	6.13	< 10^{-16}
CV_2452		Linoleoyl-CoA desaturase	2.49	< 10^{-16}
CV_4378		Probable phosphatidylethanolamine N-methyltransferase	4.62	< 10^{-16}
		**Iron metabolism**		
CV_0895	*oprC*	Iron complex outermembrane recepter protein	3.34	< 10^{-16}
CV_1452		Probable iron-sulfur 4Fe-4S ferredoxin transmembrane protein	4.24	< 10^{-16}
CV_1699		Probable tonB-dependent receptor	7.89	< 10^{-16}
CV_3553	*feoB*	Ferrous iron transport protein B	5.05	< 10^{-16}
		**Hypothetical Proteins**		
CV_0333		Conserved hypothetical protein	6.21	< 10^{-16}
CV_0334		Conserved hypothetical protein	2.85	< 10^{-16}
CV_0336		Conserved hypothetical protein	2.74	< 10^{-16}
CV_0521		Probable membrane protein	8.16	< 10^{-16}
CV_0758		Hypothetical protein	4.32	< 10^{-16}
CV_1004		Hypothetical protein	2.57	< 10^{-16}
CV_1142		Hypothetical protein	3.09	< 10^{-16}
CV_1727		Conserved hypothetical protein	4.58	< 10^{-16}
CV_1833		Conserved hypothetical protein	2.43	< 10^{-16}
CV_1903		Conserved hypothetical protein	3.50	< 10^{-16}
CV_2002		Hypothetical protein	4.94	< 10^{-16}

CV_2189	Conserved hypothetical protein	4.07	$< 10^{-16}$
CV_2383	Hypothetical protein	3.32	$< 10^{-16}$
CV_2448	Conserved hypothetical protein	3.86	$< 10^{-16}$
CV_2450	Hypothetical protein	6.80	$< 10^{-16}$
CV_2531	Probable transmembrane protein	6.26	$< 10^{-16}$
CV_2695	Hypothetical protein	6.79	$< 10^{-16}$
CV_2696	Conserved hypothetical protein	6.21	$< 10^{-16}$
CV_3155	Conserved hypothetical protein	2.75	$< 10^{-16}$
CV_3191	Conserved hypothetical protein	2.77	$< 10^{-16}$
CV_3662	Hypothetical protein	3.16	$< 10^{-16}$
CV_3867	Hypothetical protein	7.90	$< 10^{-16}$
CV_3868	Conserved hypothetical protein	7.93	$< 10^{-16}$
CV_4085	Conserved hypothetical protein	2.89	$< 10^{-16}$
CV_4086	Conserved hypothetical protein	2.68	$< 10^{-16}$
CV_4322	Conserved hypothetical protein	2.86	$< 10^{-16}$
CV_4323	Conserved hypothetical protein	2.96	$< 10^{-16}$

*Genes shared between our both microarrays

Table S3 List of genes shared among our microarray analyses and the CHP stimulon

**WTCHP/WT**	**Δ*osbR*/WT**	**Δ*osbR*(pJN105)/WT(*osbR*)**	**Gene**	**Function**
67196_3489		67196_3489		Hypothetical protein
CV_0636	CV_0636			Hypothetical protein
CV_0993	CV_0993			MFS transporter, AAHS family, 4-hydroxybenzoate transporter
CV_0118		CV_0118	*gltK*	Glutamate/aspartate transport system permease protein
CV_0190		CV_0190		Glyceraldehyde 3-phosphate dehydrogenase
CV_0209		CV_0209	*ohrA*	
CV_0210		CV_0210	*ohrR*	
CV_0398		CV_0398	*exbD2*	Biopolymer transport protein
CV_0399		CV_0399	*exbB*	Biopolymer transport protein ExbB
CV_0400		CV_0400		Periplasmic protein TonB
CV_1105		CV_1105	*gltS*	Sodium/glutamate symport carrier protein
CV_1137		CV_1137	*adhE*	Acetaldehyde dehydrogenase
CV_1174		CV_1174		Cytochrome-c oxidase, subunit I
CV_1481		CV_1481	*aroF*	2-dehydro-3-deoxy-phosphoheptonate aldolase
CV_1482		CV_1482	*cbaA* (*entA*)	2,3-dihydro-2,3-dihydroxybenzoate dehydrogenase
CV_1483		CV_1483	*cbaB* (*entB*)	Isochorismatase

CV_1484		CV_1484	*cbaE* (*entE*)	2,3-dihydroxybenzoate-AMP ligase
CV_1485		CV_1485	*cbaC* (*entC*)	Isochorismate synthase EntC / MenF
CV_1486		CV_1486	*cbaF* (*entF*)	Enterobactin synthetase component F
CV_1934		CV_1934	*metY*	O-acetylhomoserine (thiol)-lyase
CV_2446		CV_2446		3-oxoacyl-[acyl-carrier-protein] synthase III
CV_2449		CV_2449		Coenzyme F390 synthetase
CV_2540	CV_2540	CV_2540	*narI*	Nitrate reductase
CV_2541	CV_2541	CV_2541	*narJ*	Nitrate reductase molybdenum cofactor assembly chaperone
CV_2542	CV_2542	CV_2542	*narH*	Nitrate reductase / nitrite oxidoreductase, beta subunit
CV_2543	CV_2543	CV_2543	*narG*	Nitrate reductase / nitrite oxidoreductase, alpha subunit
CV_2695		CV_2695		Hypothetical protein
CV_2696		CV_2696		Hypothetical protein
CV_2970		CV_2970		Hypothetical protein
CV_3298		CV_3298	*lamB*	Maltoporin precursor
CV_3566		CV_3566		Hypothetical protein
CV_4002		CV_4002	*glnK*	Nitrogen regulatory protein P-II-2
CV_4003		CV_4003	*amtB*	Ammonium transporter
CV_4084		CV_4084		Probable protease

CHP results from a previous work [30]. Blue and orange colors indicate genes activated or repressed by OsbR, respectively.
